# Budding Yeast Rif1 Controls Genome Integrity by Inhibiting rDNA Replication

**DOI:** 10.1371/journal.pgen.1006414

**Published:** 2016-11-07

**Authors:** Maksym Shyian, Stefano Mattarocci, Benjamin Albert, Lukas Hafner, Aleksandra Lezaja, Michael Costanzo, Charlie Boone, David Shore

**Affiliations:** 1 Department of Molecular Biology, University of Geneva, Geneva, Switzerland; 2 Institute of Genetics and Genomics in Geneva (iGE3), Geneva, Switzerland; 3 University of Toronto, Donnelly Centre, Toronto, Ontario, Canada; IFOM, ITALY

## Abstract

The Rif1 protein is a negative regulator of DNA replication initiation in eukaryotes. Here we show that budding yeast Rif1 inhibits DNA replication initiation at the rDNA locus. Absence of Rif1, or disruption of its interaction with PP1/Glc7 phosphatase, leads to more intensive rDNA replication. The effect of Rif1-Glc7 on rDNA replication is similar to that of the Sir2 deacetylase, and the two would appear to act in the same pathway, since the *rif1Δ sir2Δ* double mutant shows no further increase in rDNA replication. Loss of Rif1-Glc7 activity is also accompanied by an increase in rDNA repeat instability that again is not additive with the effect of *sir2Δ*. We find, in addition, that the viability of *rif1Δ* cells is severely compromised in combination with disruption of the MRX or Ctf4-Mms22 complexes, both of which are implicated in stabilization of stalled replication forks. Significantly, we show that removal of the rDNA replication fork barrier (RFB) protein Fob1, alleviation of replisome pausing by deletion of the Tof1/Csm3 complex, or a large deletion of the rDNA repeat array all rescue this synthetic growth defect of *rif1Δ* cells lacking in addition either MRX or Ctf4-Mms22 activity. These data suggest that the repression of origin activation by Rif1-Glc7 is important to avoid the deleterious accumulation of stalled replication forks at the rDNA RFB, which become lethal when fork stability is compromised. Finally, we show that Rif1-Glc7, unlike Sir2, has an important effect on origin firing outside of the rDNA locus that serves to prevent activation of the DNA replication checkpoint. Our results thus provide insights into a mechanism of replication control within a large repetitive chromosomal domain and its importance for the maintenance of genome stability. These findings may have important implications for metazoans, where large blocks of repetitive sequences are much more common.

## Author Summary

Rif1 is a conserved eukaryotic protein implicated in regulation of both the temporal pattern of DNA replication initiation and the DNA damage response (DDR). We found that in budding yeast several of Rif1’s DDR-related phenotypes stem from its ability to interact with the Glc7/PP1 phosphatase and inhibit DNA replication initiation at the highly repetitive and highly transcribed rDNA locus. Each rDNA copy contains a potential replication origin flanked on one side by a proteinaceous replication fork barrier, a crucial player in rDNA array size maintenance. Additionally, the rDNA RFB ensures that replication proceeds in the same direction as transcription, thus presumably minimizing collisions between the replication and transcription machineries. Our results show that inhibition of rDNA origin firing by Rif1-Glc7/PP1 prevents the buildup of an excess of stalled forks within the rDNA locus, which can lead to genome instability and cell death. These findings highlight the challenges posed by the replication of repetitive loci, and in particular the need to limit DNA replication initiation events at such vulnerable regions. Our study may have important implications for metazoan genomes, which contain a much higher fraction of repetitive sequences than budding yeast. Finally, since tumor cells already exhibit elevated levels of replication stress, our results suggest that inhibition of systems that limit DNA replication initiation may jeopardize the viability of these cells and thus prove to be a useful therapeutic strategy.

## Introduction

In eukaryotes DNA replication initiates from multiple sites (origins) in a characteristic sequential pattern referred to as ‘replication timing’ [1]. Replication timing is tightly regulated, with some origins being replicated early during S phase and others later [2, 3]. Mechanisms that determine replication timing are still unclear, though recent studies point to a model in which limiting factors (e.g. Sld3, Sld2, Dpb11, Dbf4) are sequentially re-distributed to origins with decreased levels of accessibility to these factors, thus generating a temporal program of origin firing [4, 5].

**Fig 1:**
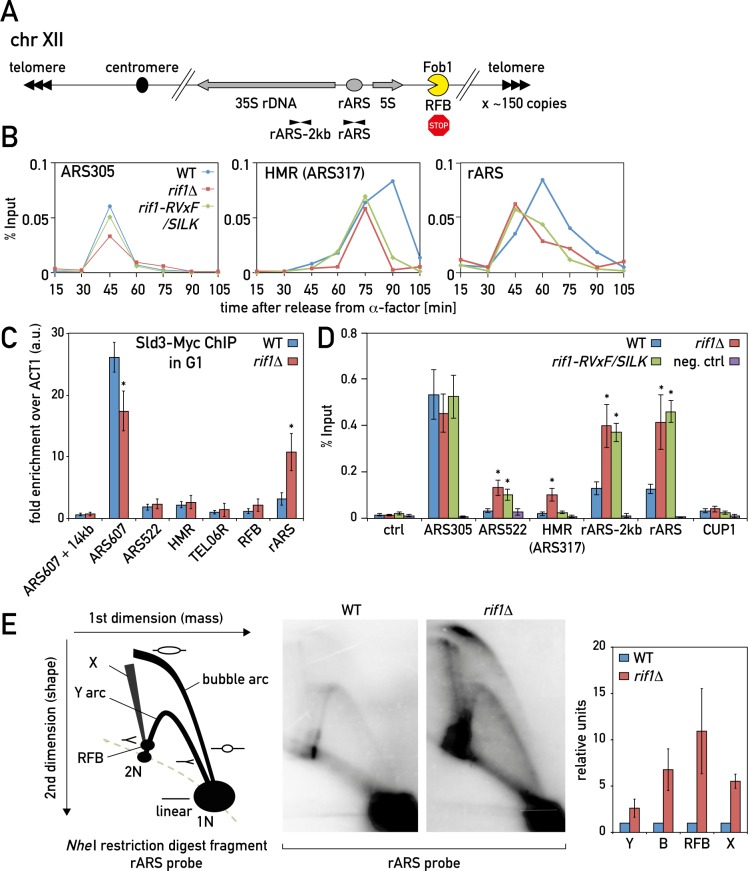
Rif1-Glc7 inhibits replication initiation at the rDNA locus. (A) Schematic representation of budding yeast chromosome XII and the rDNA repeat array. “rARS” and “rARS-2Kb” indicate the position of primers used for the BrdU-IP-qPCR experiments. (B) Leading strand DNA polymerase (Pol2-13xMyc) ChIP-qPCR in wild type (WT), *rif1Δ* and *rif1-RVxF/SILK* cultures synchronously released into S phase at 18°C from alpha-factor arrest. (C) Sld3-13xMyc ChIP-qPCR in G1 arrested cells. (D) BrdU incorporation at the indicated loci in WT, *rif1Δ* and *rif1- RVxF/SILK* mutant cultures released from nocodazole (G2/M) arrest into 0.2 M HU for 2 hrs. The negative control strain (neg. ctrl) lacks the BrdU incorporation cassette. Data are presented as mean +/- SEM of three independent experiments and a t-test was used to compare the means of WT and mutant cultures. (*) P < 0.05. (E) 2D agarose gel electrophoresis of *Nhe*I-digested genomic DNA, Southern blotted and probed with rARS probe (see scheme in S1D Fig) from WT and *rif1Δ* cultures arrested and released as in (D). The left panel is a schematic diagram depicting possible replication intermediates; the middle panel is representative images; the right panel is quantification of the n = 2 experiments. Y–Y arc, B–bubble arc, X–X-shaped molecules, corresponding to almost fully replicated molecules, recombination intermediates and Holliday junctions. See also S1 Fig.

In the budding yeast *Saccharomyces cerevisiae* about one-third of all potential replication origins (autonomously replicating sequences, or ARSs) are located within the rDNA repeat array on chromosome XII [6]. The rDNA array comprises ~150–200 copies of a module containing 35S and 5S rRNA genes separated by two intergenic regions harboring an origin of replication (rARS) and a polar replication fork barrier (RFB) (Fig 1A). Interestingly, only ~20% of the rDNA origins fire during a given S phase in wild type cells [6, 7], and it has been shown that deregulation of rDNA replication leads to genomic instability [8, 9].

The rDNA repeat RFB, which is generated by sequence-specific binding of the Fob1 protein [10], is believed to prevent head-to-head collisions of the transcription and replication machineries and to mediate rDNA copy number homeostasis. Replication fork blockage caused by other proteins is observed elsewhere in the genome, for example at tRNA promoters, telomeres, silent mating type loci, dormant origins and centromeres [11–13]. The replisome protection (or pausing) complex, which consists of Tof1/Csm3 in *S*. *cerevisiae*, is essential for fork arrest at RFBs both within and outside of the rDNA [13, 14] and is proposed to act mainly by counteracting the Rrm3 helicase [14]. Consistent with this idea, genome-wide accentuation of RFBs in *rrm3Δ* cells leads to fork collapse and breakage, and to loss of viability in combination with mutation of DNA repair genes such as *MRE11*, *SGS1*, and *SRS2* [15, 16]. Similarly, specific strengthening of the rDNA RFB by Fob1 overexpression decreases viability in *mre11Δ* mutants [17]. Apart from its conserved function in DNA double strand break (DSB) nucleolytic processing (resection) [18] the MRX complex (Mre11, Rad50 and Xrs2) also participates in replication fork stabilization under conditions of replication stress (e.g. dNTP depletion; [19]) and in replisome re-assembly after fork collapse [20].

Repair of broken replication forks at the yeast rDNA RFB leads to repeat array instability due to recombination-driven gain or loss of copies [10]. Accordingly, an increase in the efficiency of rDNA origin firing, such as that observed in cells mutated for the histone deacetylase Sir2 [6, 7], is associated with elevated rDNA instability [8, 9, 21], presumably due to an increase in the number of replication forks arrested at an RFB. One model suggests that, in addition to down-regulating rDNA origin firing, Sir2 also inhibits unequal sister chromatid exchange, by promoting cohesion binding within the rDNA intergenic spacers [22], thus defining a second mechanism by which Sir2 promotes rDNA stability.

Rif1, a budding yeast Rap1-interacting factor, was initially described as an inhibitor of telomerase-dependent lengthening of telomeres in yeast [23]. Rif1 is highly conserved in eukaryotes [24, 25] and has more recently been shown to be a regulator of DNA replication initiation in yeast, flies and mammals [26–33]. We and others found that Rif1, through its conserved RVxF/SILK motifs, interacts with protein phosphatase 1 (PP1; Glc7 in budding yeast), and that this interaction is crucial for inhibition of replication origin firing by counteracting the activity of the Dbf4-dependent kinase (DDK) [26, 27, 30]. Deletion of *RIF1* in budding yeast leads to advancement of the replication timing of most late origins [28]. Importantly, loss of Rif1 in mouse cells leads to defects in S phase progression, hypersensitivity to the DNA polymerase inhibitor aphidicolin, and checkpoint kinase activation [31, 34]. Modulation of Rif1 activity may thus provide a valuable tool to study the molecular and cellular consequences of altering the replication-timing program.

Here we show that budding yeast Rif1 inhibits DNA replication initiation at the rDNA locus and thus promotes the stability of rDNA repeat array. Moreover, the increase of rDNA instability in *rif1Δ* accounts for the majority of its DNA damage response (DDR)-related phenotypes, suggesting that the rDNA is a key target of Rif1 action. These findings offer a new perspective on the relationship between replication timing, repeated DNA sequences and genome stability.

## Results

### Rif1 inhibits replication initiation at the rDNA locus through its interaction with the PP1 phosphatase Glc7

Driven by the hypothesis that disruption of *RIF1* leads to an increase in the number of active replication forks during S phase [26–28, 30], we decided to determine where these forks are located. Chromatin immunoprecipitation (ChIP) of epitope-tagged DNA polymerase epsilon (Pol2) from cell cultures synchronously entering S phase provides a read-out of replication fork passage [26, 27, 35]. We thus measured Pol2 association at different loci, comparing wild type to *rif1* mutants. Consistent with our previous observations [27], the timing of Pol2 recruitment to the early origins was not affected in cells deleted for *RIF1* or cells mutated in its Glc7-binding RVxF/SILK motifs (see *ARS305* at the Fig 1B, where in both WT and the *rif1* mutants Pol2 is recruited at 45 minutes after the release into S phase). On the other hand, Pol2 was detected over a shorter time interval at the late replicating *HMR* locus in both *rif1* mutants, which might reflect its earlier and/or faster replication. Given the fact that many dormant replication origins are located within the repetitive rDNA locus [6, 7], we also probed for the ARS element there (referred to as rARS). In wild type cells rARS displayed a peak of Pol2 binding in the middle of S phase (60 minutes following release from alpha-factor arrest). Interestingly, deletion of *RIF1* or mutation of its Glc7 (PP1)-interacting RVxF/SILK motifs advanced Pol2 binding by ~15 minutes, to a time similar to that of the early *ARS305* origin. Importantly, acute depletion of Rif1 from the nucleus in G1 phase by the anchor-away method (see Materials and Methods section for details) also led to advancement of Pol2 binding in the next S phase at rARS, *HML* and telomere sites, while replication timing of an early origin (*ARS607*) was not affected (S1A Fig, left and middle panels).

Origins that fire early in S phase, but not late-replicating regions, recruit the pre-replicative complex (pre-RC) component Sld3 in G1, prior to DNA replication initiation [36]. Significantly, we detected elevated Sld3 recruitment to the late replicating rARS in G1-arrested *rif1Δ* cells (Fig 1C), whereas the recruitment of Mcm4, part of the replicative helicase that is loaded synchronously on all origins, was not affected (S1A Fig, right panel). The decrease in Sld3 recruitment to a non-rDNA early origin (*ARS607*, Fig 1C) in *rif1Δ* cells might be due to re-localization of limiting amounts of this protein to the excess of activated late origins, both within the rDNA and elsewhere, due to the absence of Rif1. Taken together, these data indicate that Rif1’s interaction with the PP1 phosphatase (Glc7) is responsible for inhibition of rDNA replication, defining the rDNA locus as a novel Rif1-Glc7 target.

To further investigate the role of Rif1 in rDNA replication, we used bromo-deoxyuridine (BrdU) incorporation followed by anti-BrdU immunoprecipitation (IP) and quantitative PCR (qPCR) as a more direct method to measure newly synthesized DNA. We released G2/M arrested (nocodazole-treated) cells into S phase in the presence of 0.2 M hydroxyurea (HU) and BrdU (see FACS profiles in S1B Fig). HU slows fork progression and allows one to determine whether late origins fire, or are instead passively replicated by forks coming from nearby early origins. In accord with a recent genome-wide study [28], we detected higher levels of DNA synthesis in *rif1Δ* at late origins (*ARS1212*, *ARS522*, *HMR* locus and telomeres), whereas levels of BrdU incorporation at early origins (*ARS305*, *ARS607*) were not affected (Figs 1D and S1C). We also found higher BrdU incorporation in *rif1Δ* and *rif1-RVxF/SILK* mutant cells compared to wild type at and around rARS (Fig 1D). Importantly, the increased BrdU incorporation in *rif1* mutants was specific to the rDNA and not a general feature of repetitive loci, since another repetitive locus (*CUP1*) incorporated BrdU very similarly in *rif1Δ*, *rif1-RVxF/SILK* and wild type cells. Analysis of the source data from the Peace et al. study [28] also revealed the same trend of higher BrdU incorporation at rARS in *rif1Δ* compared to wild type.

Next, we used 2D agarose gels [37] to observe directly the replication intermediates at the rDNA locus, again from cells released from a G2/M arrest into S phase in the presence of HU. Deletion of *RIF1* led to a dramatic increase in bubble arc, Y arc, RFB spot, and X-shaped molecules signals at the rDNA in these conditions (Figs 1E and S1D), indicating a higher frequency of rARS firing. The effect of *rif1Δ* as seen in asynchronous cultures was less prominent (S1E and S1F Fig), presumably because the increase in fork density in the mutant also increases the rate at which blocked forks are resolved following arrival of a downstream fork moving in the opposite direction, which would convert the replication intermediates into linear molecules. This effect is nullified when synchronized cells are released into S phase in medium containing HU, which permits early origin firing but severely limits fork elongation. We thus conclude that the realm of Rif1-dependent inhibition of DNA replication initiation includes the rDNA locus.

### Rif1-Glc7 acts together with Sir2 to inhibit rARS firing, but independently at non-rDNA late origins

**Fig 2:**
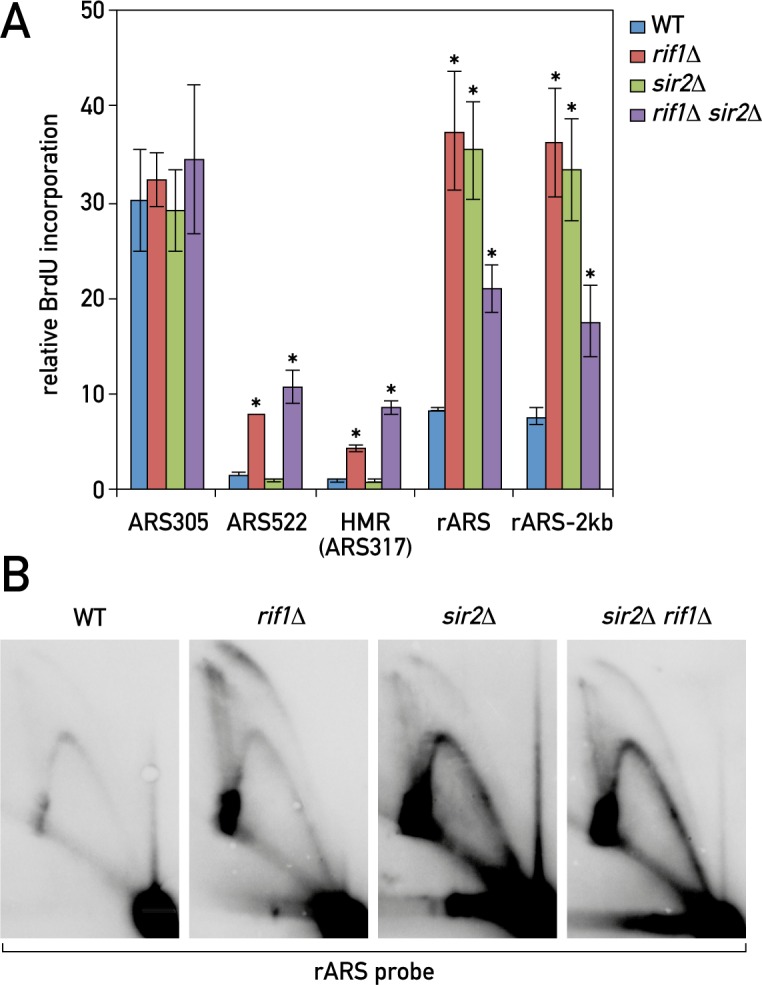
Rif1 acts together with Sir2 to inhibit rDNA origin firing, but independently to block late origin firing. (A-B) Relative BrdU incorporation at the indicated loci (A) and 2D agarose gel electrophoresis (B) in WT, *rif1Δ*, *sir2Δ*, and *rif1Δ sir2Δ* mutant cultures released from nocodazole (G2/M) arrest into 0.2 M HU for 2 hrs. Data are presented as mean +/- SEM of three independent experiments and a t-test was used to compare the means of WT and mutant cultures. (*) P < 0.05.

As pointed out above, Sir2 plays an important role in several aspects of rDNA biology. We confirmed the previous observation [7] of an rDNA replication increase (as detected by BrdU incorporation) upon deletion of *SIR2*, and furthermore found that Sir2’s effect on rARS is quantitatively similar to that of Rif1 (Fig 2A). However, unlike *rif1Δ* cells, *sir2Δ* mutants do not display increased firing at either of the two late-replicating regions we examined, *ARS522* or *HMR* (*ARS317*) (Fig 2A). To address the question of whether Rif1 acts independently of Sir2 to inhibit rARS firing, we examined a *rif1Δ sir2Δ* double mutant, but found no additive effect, suggesting that these two proteins act in a common pathway. In fact, and quite surprisingly, the *rif1Δ sir2Δ* double mutant displayed consistently lower BrdU incorporation at both rARS and a site 2 kb distant, compared to both single mutants. Nevertheless, replication at these sites was still increased at least 2-fold over that observed in wild type cells.

Using 2D gels we also observed more intensive replication and fork pausing in early S phase at the rDNA in *sir2Δ* cultures, similar to that in *rif1Δ* (Fig 2B), though with a marked difference in the relative intensity of the arc signals. Deletion of *RIF1* mostly increased bubble arcs, whereas *SIR2* deletion and the double deletion of *SIR2* and *RIF1* led to more Y arcs. This difference might be due to variations in the spatial pattern of origin activation, fork progression rates, or timing of origin activation in these mutants In conclusion, the above results indicate that Sir2 and Rif1 work in a common pathway to inhibit rARS firing, but suggest in addition that other players may be involved that create a more complex functional relationship between Sir2 and Rif1 (see Discussion).

### Rif1-Glc7 acts to diminish RFB-induced rDNA instability

As indicated above, replication of the rDNA repeats is highly polar in nature due to an orientation-dependent replication fork block (RFB; see Fig 1A). Replication proceeding rightwards from rARS is efficiently blocked at the RFB, which is thought to prevent potential collisions with an RNA polymerase I (RNAPI) complex transcribing the downstream copy of the 35S rRNA gene. Forks proceeding to the left from rARS, and in the direction of 35S rRNA gene transcription, are free to pass the RFB present at the upstream rDNA copy.

**Fig 3:**
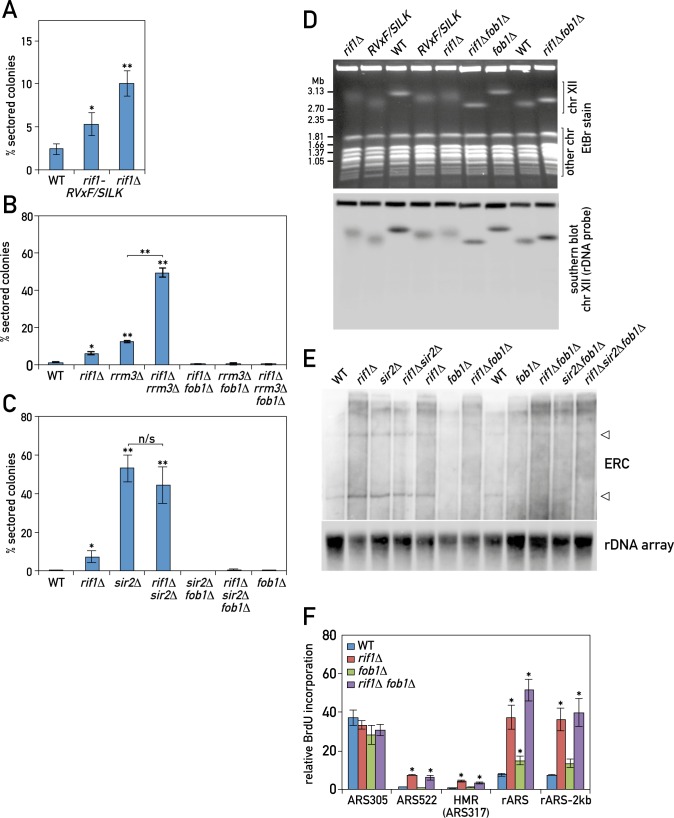
Rif1 acts together with the Glc7/PP1 phosphatase to diminish Fob1/RFB-induced rDNA instability. (A-C) rDNA instability for the indicated WT and mutant strains was measured by the *ADE2* marker-loss assay. (D) The chromosomes (chr) from indicated strains were resolved using pulsed-field gel electrophoresis (PFGE) and stained with ethidium bromide (top panel). The same gel was rDNA probed and Chr. XII was detected by Southern blot (bottom panel). Note that the size of Chr. XII is variable even in WT strains due to the presence of the rDNA locus [38]. (E) Southern blot of undigested genomic DNA from the indicated strains reveals ERC accumulation (upper panel). The same samples were loaded on an agarose gel for the quantification of total rDNA amounts (lower panel). (F) Relative BrdU incorporation in cells released from nocodazole-induced G2/M arrest into medium containing 0.2 M HU. Data are presented as in Fig 1. (*) P < 0.05; (**) P <0.01. See also S2 and S3 Figs.

We hypothesized that rDNA locus stability might be sensitive to an increase in origin firing since this leads to a concomitant increase in the number of forks blocked at RFBs (Fig 1E, Fig 2B). Blocked forks can, with a certain probably, collapse, sometimes generating DNA breaks that will normally be repaired by homologous recombination (HR), non-homologous end joining (NHEJ) or alternative break-induced replication (BIR) pathways. Due to the repetitive nature of the rDNA, recombination between different repeats of the same or sister chromatids may lead to a change in the rDNA array size, which is usually referred as ‘rDNA instability’ [38]. The loss of repeats from the rDNA array can be conveniently measured when a single copy of the *ADE2* gene is inserted in the array, in cells where the endogenous *ADE2* gene is mutated. The *ADE2* gene confers a white colony-color phenotype, whereas popping-out of this gene from the chromosome (together with adjacent repeats) leads to the accumulation of a red pigment when adenine in the medium is limiting, and the appearance of red sectors in colonies [39]. Indeed, using this colony-color marker-loss assay [39], we detected higher levels of rDNA instability in *rif1Δ* cells compared to wild type (Figs 3A and S2A), consistent with a recent report [40].

To further challenge the idea that the rDNA instability phenotype of *rif1Δ* is specifically linked to its effect on replication origin firing, we examined the *rif1-RVxF/SILK* mutant, which we showed previously [27] to result in a loss of the Rif1-Glc7 interaction and increased phosphorylation of two key DDK kinase targets at pre-RCs. As shown above, the *rif1-RVxF/SILK* mutant leads to increased and earlier rDNA origin firing (Fig 1B and 1D). We found that *rif1-RVxF/SILK* mutant cells also display a higher level of rDNA instability compared to wild type (Fig 3A), though smaller than the increase conferred by complete deletion of *RIF1*, perhaps because rif1-RVxF/SILK retains some residual binding to Glc7 [27].

We next hypothesized that strengthening of the RFB by deletion of *RRM3* [15, 16], which encodes a helicase that promotes the passage of replication forks through RFBs [41], would lead to a further increase in rDNA instability. As predicted, we observed an additive increase in rDNA instability when combining *rif1Δ or rif1-RVxF/SILK* with *rrm3Δ* (Figs 3B, S2A and S2D). If the effect of Rif1 and Rrm3 on rDNA stability were linked to the RFB, deletion of the *FOB1* gene, whose product is required to establish the fork block, would be expected to abolish the instability induced by *rif1* mutants, *rrm3Δ*, or the double mutants *rif1Δ rrm3Δ* and *rif1-RVxF/SILK rrm3Δ*. This is indeed what we found (Figs 3B, S2A and S2D), strongly suggesting that Rif1, as well as Rrm3, act through the RFB. Surprisingly, neither single mutation (*rif1Δ* or *rrm3Δ*) nor the double mutation *rif1Δ rrm3Δ* affected cell growth, either under normal conditions or in the presence of DNA damaging agents (S2C Fig), suggesting that DNA repair pathways in these cells are sufficient to cope with the increased load of stalled forks [15, 16].

In accordance with its rDNA replication phenotype (Fig 2), *sir2Δ* also displays a large increase in rDNA instability that is fully rescued by *FOB1* deletion (Figs 3C and S2B). The increase in rDNA instability caused by *sir2Δ* is larger than that of *rif1Δ* and is unaffected by *rif1Δ*, consistent with a previous report [40]. Increased instability of the rDNA locus leads to heterogeneity in the size of chromosome XII in a population of the cells [22]. As expected, then, pulse field gel electrophoresis revealed a heightened smearing (broader and less sharp band) of chromosome XII in *rif1-RVxF/SILK* and *rif1Δ* cells (Fig 3D), though not to the same extent as in *sir2Δ* (S3A Fig), consistent with their varying effect on rDNA stability measured by the sectoring assay. Again as expected, we found that the effect of *rif1Δ* on chromosome XII heterogeneity was reversed by the *fob1Δ* mutation (Fig 3D). Deletion of either *RIF1* or *RRM3* increases rDNA instability (Figs 3B and S3C), but only *rrm3Δ* leads to an increase in the ratio of Fob1-dependent blocked forks at the RFB to total forks at rDNA [41] (compare 2D gels in S1E and S3C Figs), since in *rif1Δ* the increase in RFB signal is paralleled by an increase in the number of forks at the rDNA (Figs 1E and 2B). These findings further support the argument that Rrm3 acts directly at RFBs, whereas Rif1 primarily acts through controlling DNA replication initiation.

Elevated blockage and collapse of replication forks at the rDNA may also lead to HR-dependent “popping-out” of rDNA repeats in the form of episomal circles [42], referred to as extrachromosomal rDNA circles (ERCs). Consistent with elevated rDNA array instability, we observed increased levels of ERCs in *rif1Δ*, *rrm3Δ* and *sir2Δ* cells (Figs 3E and S3B). It is not known whether the rARS is more or less active on the episomal ERCs, but it is conceivable that the change in ERC number in a cell may affect the apparent rDNA replication phenotype. Deletion of *FOB1*, which has been shown to significantly reduce ERC formation ([83]; Figs 3E and S3B) abolished ERC accumulation in *rif1Δ* and *sir2Δ* mutants (Fig 3E). However, we found that *fob1Δ* did not affect the *rif1Δ*-induced increase in rDNA replication, as detected by BrdU incorporation and 2D gels (Figs 3F and S3D), confirming that the loss of Rif1 influences chromosomal rDNA origin firing. Taken together, these results show that Rif1 and Sir2, but not Fob1, are involved in control of replication initiation at the rDNA locus.

### Rif1 promotes rDNA stability through a mechanism independent of Sir2 re-localization

**Fig 4:**
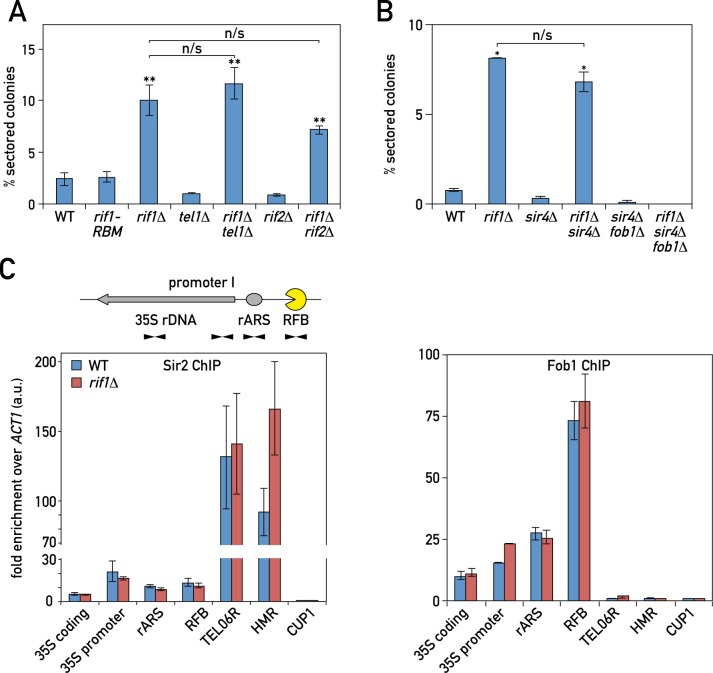
Rif1 promotes rDNA locus stability through a mechanism independent of Sir2 re-localization. (A-B) rDNA instability for the indicated WT and mutant strains was measured by the *ADE2* marker-loss assay. (C) ChIP-qPCR experiments in strains expressing Fob1-TAP or Sir2-TAP proteins. The location of the rDNA primers is indicated on the schematic above the graphs. Data are presented as mean +/- SEM and a t-test was used to compare the means of WT and mutant cultures. (*) P < 0.05. See also S4 Fig.

Rif1 was originally identified as a telomere-binding protein involved in TG-tract length regulation. Deletion of *FOB1* did not affect *rif1Δ*-dependent telomere elongation (S4A Fig), arguing that telomere- and rDNA-related functions of Rif1 are separable. However, early studies [45,47,84,85] indicated that Rif1 can compete with SIR proteins for binding to the Rap1 C-terminus at telomeres and that this competition can indirectly affect the availability of SIR proteins for binding elsewhere in the genome, in particular at silent mating type loci (where a Sir2/3/4 complex assembles) and within the rDNA, where Sir2 binds at two distinct sites. A more recent report thus suggested that *rif1Δ* increases rDNA instability indirectly by favoring the re-localization of Sir2 from its binding sites in rDNA to telomeres and silent mating type loci [40]. To determine whether Rif1 acts directly to affect rDNA stability, or instead works by modulating the distribution of Sir2 at its different target sites (rDNA, *HM* loci and telomeres), we first assessed rDNA instability in the *rif1-RBM* mutant, which, like *rif1Δ*, leads to an increase in telomeric silencing and telomere TG-tract length [43]. We found that *rif1-RBM* has no effect on rDNA stability (Fig 4A)**,** suggesting that increased SIR-mediated telomeric silencing and telomeric TG tract length do not lead to rDNA instability. Furthermore, neither deletion of *TEL1*, which reduces telomere length in a *rif1Δ* background [44], nor deletion of *RIF2*, which further increases telomere length and telomeric silencing [45], had any effect on *rif1Δ-*promoted rDNA instability (Fig 4A). We also examined *sir4Δ* cells, where the Sir2 protein cannot be recruited to either telomeres or *HM* loci and is thus liberated for enhanced action within the rDNA [46]. However, *sir4Δ* had no significant effect on *rif1Δ*-induced rDNA instability (Fig 4B). Taken together, these findings do not support the notion that Rif1 affects rDNA stability by influencing Sir2 distributions in the nucleus, but are instead consistent with Rif1 having a direct effect on rDNA stability.

Next, we measured binding of Sir2 to chromatin by ChIP-qPCR in *rif1Δ* cells. We found that Sir2 binding was increased at the *HMR* silent mating-type locus (Fig 4C, left panel), in line with the idea that Rif1 competes with the SIR complex for Rap1 binding at *HMR* in wild type cells [47]. However, in contrast to a recent report [40] that found a small effect of *rif1Δ* on Sir2 binding at IGS1 (near the RFB) using a semi-quantitative ChIP assay, we found no difference in Sir2 binding there by ChIP-qPCR, nor at three other sites along the rDNA locus: at rARS (which is located in IGS2), at an adjacent region at the 35S rRNA gene promoter, and at a site within the 35S rRNA gene coding sequence (Fig 4C, left panel). Fob1 ChIP at rDNA was also unaffected by *rif1Δ* (Fig 4C, right panel). Taken together, these data suggest that any influence of Rif1 on SIR complex distribution is insufficient to account for its effects on rDNA instability, and instead argue that Rif1 has a direct effect on rDNA stability by maintaining the low level of rARS firing.

Martina et al. [48] recently proposed that Rif1 physically counteracts Rad9 binding to DSBs. We therefore asked whether *rif1Δ*-induced rDNA instability stems from unrestrained activity of Rad9. However, deletion of *RAD9* alone had no effect on rDNA instability in the marker-loss assay and did not alleviate the increased instability caused by *rif1Δ* (S4B Fig). We therefore conclude that the effect of *rif1Δ* on rDNA stability is unrelated to the activity of Rad9. Since the *rad9Δ* mutation abolishes the DNA damage checkpoint (DDC) [49], these results also argue that *rif1Δ*-dependent elevation in rDNA instability is not a consequence of DDC activation.

### rDNA instability, driven by increased replication and fork stalling, is the major cause of synthetic lethality in *rif1Δ* cells

Arrested replication forks need to be stabilized and/or restarted to avoid formation of DSBs and/or inappropriate recombination events [13]. Increased numbers of stalled replication forks might therefore compromise cell viability. Consistent with this idea, elevating the strength of RFBs, either by removal of the Rrm3 helicase or by overexpression of Fob1, leads to synthetic sickness in combination with disruption of the MRX complex [15–17], probably due to a role for MRX in fork repair [50], fork restart [20], or fork stabilization at RFBs [17].

**Fig 5:**
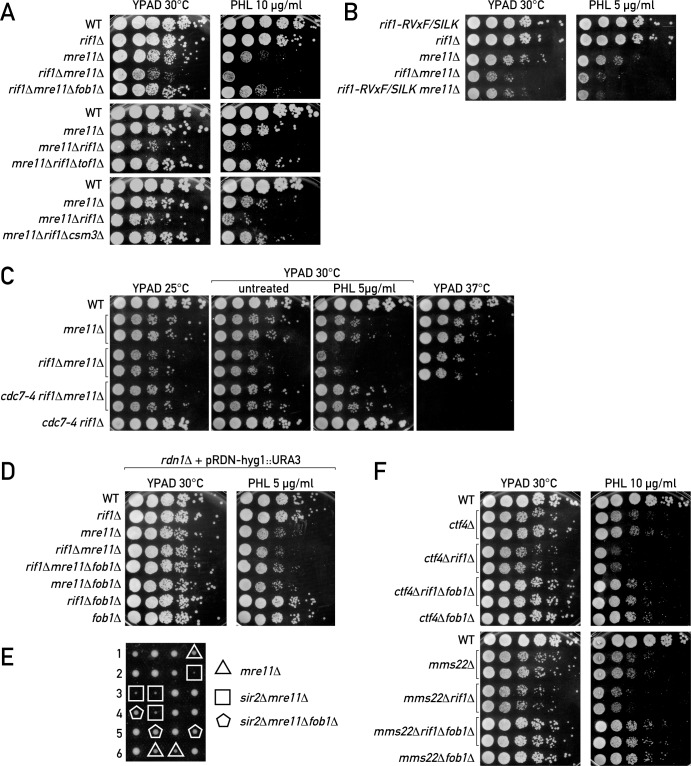
rDNA instability is the major cause of synthetic lethality in *rif1Δ* cells. Overnight cultures of the indicated genotypes were serially diluted 1:10 and spotted onto solid YPAD medium or YPAD medium supplemented with phleomycin (PHL). Plates were incubated at the indicated temperature for 2–4 days before being photographed. (A) The synthetic sickness of *rif1Δ mre11Δ* cells is rescued by deletion of the *FOB1* gene. (B) The *rif1 RVxF/SILK* mutant is synthetic sick with *mre11Δ* both in normal conditions and in the presence of PHL. (C) Effect of the *cdc7-4* allele on synthetic sickness of *rif1Δ mre11Δ* mutants. (D) Effect of *rdn1Δ* on synthetic interactions of *mre11Δ* and *rif1Δ*. (E) Tetrad dissection showing the synthetic sickness of *sir2Δ mre11Δ* and its rescue by *fob1Δ*. (F) Synthetic sickness of *rif1Δ* with *ctf4Δ* and *mms22Δ* is rescued by *fob1Δ*. See also S5 and S6 Figs.

As already reported, deletion of *RIF1* also severely compromises growth of *mre11Δ* cells, both in untreated cells and upon exposure to phleomycin, which generates DSBs (Fig 5A; [48, 51]). We reasoned that this effect of *rif1Δ* might stem, at least in part, from an increased number of replication forks that are pausing at an rDNA RFB in *rif1Δ* cells, and thus prone to collapse and subsequent DSB formation. If this were the case, deletion of *FOB1* or alleviation of the fork pause through removal of the replisome pausing complex (Tof1/Csm3) would be expected to rescue this synthetic sickness. Indeed, *fob1Δ*, *tof1Δ*, or *csm3Δ* deletions completely rescued *rif1Δ mre11Δ* synthetic sickness, both in normal conditions and upon treatment with genotoxic agents (Figs 5A, S5A and S5B).

We next examined the premise that the increased number of RFB-stalled forks in *rif1Δ mre11Δ* cells stems specifically from the effect of Rif1 on rARS firing. In support of this notion, we found that mutation of the Rif1 RVxF/SILK motifs alone conferred a synthetic sickness phenotype in combination with *mre11Δ* that was comparable to that of *rif1Δ*, whereas *rif1-RBM* had no such effect (Figs 5B and S5C). Given our finding that *rif1-RVxF/SILK*, but not *rif1-RBM*, increases rARS firing [27], these data point to a primary effect of Rif1 on rARS firing as the cause for synthetic sickness in combination with *mre11Δ*. To test this idea further, we introduced the temperature-sensitive *cdc7-4* mutation, which compromises DDK kinase activity and thus decreases replication initiation rates genome-wide [52], into our *rif1Δ mre11Δ* strain. At 30°C, where compromised cdc7-4 activity begins to affect growth in a *rif1Δ* background, we note significant alleviation of *rif1Δ mre11Δ* synthetic sickness, both in untreated and phleomycin-treated cells (Fig 5C). As expected, at 37°C *cdc7-4* is unable to support viability in either the *rif1Δ* or the *rif1Δ mre11Δ* background.

Finally, we reasoned that if elevated rDNA repeat replication coupled with fork blockage at the RFB were the source of toxicity in *rif1Δ mre11Δ* double mutants, then removing the rARS/RFB replication system from chromosome XII should improve growth of these cells. To test this idea we took advantage of a previously described rDNA array deletion strain, which leaves only 2 chromosomal rDNA repeats (*rdn1Δ* strain) [53]. Survival of this strain is maintained by a multi-copy plasmid harboring both the 35S and 5S rRNA genes but having a 2 μm replication origin instead of rARS. As predicted, this rDNA repeat- and rARS-deficient strain displayed no evidence of *rif1Δ mre11Δ* synthetic sickness, nor any effect of *FOB1* deletion (Fig 5D).

In line with its strong additive effect in combination with *rif1Δ* (Fig 3B), *rrm3Δ* also displays strong synthetic sickness with *mre11Δ*. However, consistent with a more general role of Rrm3 at replisome barriers throughout the yeast genome [41], *rrm3Δ* synthetic sickness with *mre11Δ* was more severe than that of *rif1Δ mre11Δ* and was significantly but not completely suppressed by *fob1Δ* (S6A Fig). Moreover, double deletion of *RRM3* and *RIF1* was inviable in combination with *mre11Δ*, in line with the additive effects of Rrm3 and Rif1 on rDNA integrity (S6A Fig).

Consistent with the fact that *sir2Δ*, like *rif1Δ*, leads to elevated rDNA origin usage and rDNA instability (Fig 2, Fig 3C and 3D), we found that *sir2Δ* also shows *fob1Δ*-suppressible synthetic sickness with *mre11Δ* (Fig 5E). The triple mutant *rif1Δ sir2Δ mre11Δ* was slightly less sick than *rif1Δ mre11Δ* (S6B Fig), consistent with a partial decrease in replication at the rDNA when combining *rif1Δ* with *sir2Δ* (Fig 2A and 2B). In line with our conclusion that the Rif1 effect on rDNA is independent of Sir2 re-localization, *sir4Δ* mutation did not alleviate the synthetic sickness of *rif1Δ* with *mre11Δ* (S6C Fig) as it had no significant effect on *rif1Δ*-induced rDNA instability (Fig 4B).

We next reasoned that the burden of elevated replication in *rif1Δ* should lead to a synthetic growth defect in combination with other mutations affecting replisome integrity. In fact, combining *rif1Δ* with deletion of *CTF4*, which encodes a replisome component that couples CMG helicase and DNA polymerase alpha/primase [54], led to a strong synthetic sick phenotype (Fig 5F). The same is true for deletion of *MMS22*, whose product has been proposed to be recruited by Ctf4 to the replisome [55] as part of the Rtt101-Mms1-Mms22 ubiquitin-conjugating complex, essential for replisome maintenance at endogenous impediments and upon challenge with genotoxic agents [56] (Fig 5F; [57]). It worth noting that, together with histone acetyltransferase Rtt109, Mms22 also influences the downstream repair events at blocked forks [58–60] and participates in the maintenance of rDNA array size [61]. The synthetic interaction of *rif1Δ* with both *ctf4Δ* and *mms22Δ* was also alleviated by the deletion of *FOB1*, again consistent with the idea that the primary defect occurs at the rDNA RFBs (Fig 5F). All of the above results show that the function of Rif1 in rDNA stability becomes essential for survival when replisome maintenance and/or DNA break repair at rDNA RFBs is compromised.

### Rif1 functions beyond the rDNA locus to suppress DNA replication checkpoint activation

**Fig 6:**
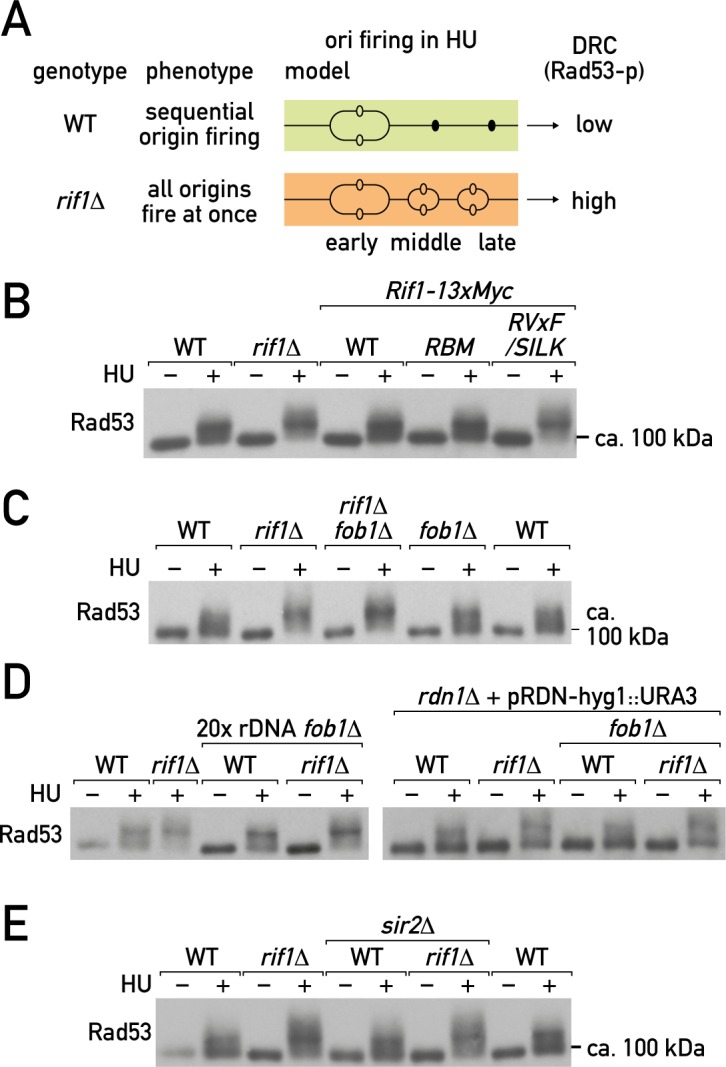
Rif1 functions in budding yeast beyond the rDNA locus. (A) A proposed model for Rif1 effects on both DNA replication timing and DNA replication checkpoint activation in budding yeast. (B-E) Western blots of total Rad53 following HU (0.2 M for 2 hrs) or mock treatment of asynchronous cultures of the indicated mutant combinations: (B) *rif1-RBM* and *rif1-RVxF/SILK*, (C) *rif1Δ* and *fob1Δ*, (D) *rif1Δ* and *fob1Δ* in strains harboring 20 or 2 (*rdn1Δ*) rDNA repeats on chromosome XII, or (E) *rif1Δ* and *sir2Δ*. See also S7 Fig.

Treatment of cells with the ribonucleotide reductase inhibitor HU leads to DNA replication checkpoint (DRC) activation due to accumulation of single-stranded DNA at stalled replication forks, and is accompanied by Rad53 phosphorylation (reviewed in ([62]). Interestingly, the strength of the DRC, as measured by Rad53 phosphorylation (Rad53-p), correlates with the number of arrested replication forks [63]. We thus reasoned that increased replication in early S phase in *rif1Δ* cells might lead to higher DRC activation (Fig 6A). Indeed, we observed reproducibly higher levels of Rad53-p following HU treatment in both *rif1Δ* cells (in the W303 background (Fig 6B–6D), and two other backgrounds, S288C and JC482 (S7A Fig, upper panels)), and in cells where Rif1 was rapidly depleted from the nucleus by anchor-away (S7A Fig, lower panel). Moreover, only the *rif1-RVxF/SILK* mutant, but not *rif1-RBM*, exhibited elevated DRC upon HU treatment (Fig 6B), emphasizing the connection with increased replication. This effect of Rif1 loss depended on the DRC adaptor Mrc1, but not the DDR adaptor Rad9 (S7B Fig), indicating that it is a *bona fide* DRC response [62]. Interestingly, the kinetics of Rad53 de-phosphorylation upon HU withdrawal (recovery from the DRC) were similar in wild type and *rif1Δ* cells (S7C Fig), suggesting that DRC deactivation is not altered by *rif1Δ*, consistent with previous observations [28]. Importantly, despite the elevated DRC, *rif1Δ* does not confer increased sensitivity to genotoxic agents such as HU, MMS, phleomycin or camptothecin (S2C and S3 Figs; [48]), suggesting that Rif1 has no essential role in the DDR, either in DSB repair, repair of damaged forks, or re-start of stalled replication forks.

Deletion of *FOB1* had no effect on either the DRC (Fig 6C) or the replication phenotype (Fig 3F) of *rif1Δ* cells, suggesting that their elevated Rad53-p might result from an increase in rDNA replication *per se* and not from subsequent fork stalling and/or breakage at the rDNA RFBs. However, we found that removal of the majority of the rDNA repeats, leaving only 20 or 2 copies [53, 64], also did not alleviate the *rif1Δ*-induced increase of Rad53-p upon HU treatment (Fig 6D). This suggests that even when a large increase in rDNA replication initiation is not possible, the effect of *rif1Δ* on replication initiation elsewhere is sufficient to lead to elevated Rad53-p, a conclusion consistent with the observation that *rif1Δ* increases origin activation near telomeres and at other sites in the genome (this report and [26–28, 30]).

It is important to note that in contrast to *rif1Δ*, deletion of *SIR2* did not increase the level of Rad53-p upon HU (Fig 6E). This might be due to the fact that in *sir2Δ* cells the increase in rARS usage is accompanied by a decreased efficiency of firing of genomic origins outside of the rDNA locus [7], whereas *rif1Δ* leads to elevation of the total replication load on the genome, due to its effects at both at rDNA and elsewhere. Thus, rDNA origins are only one example of a general Rif1 inhibitory role in DNA replication initiation. Nevertheless, due to the intrinsic vulnerability of the repetitive rDNA locus, caused at least in part by the RFB [64], Rif1’s role in genome integrity is manifested largely at this site.

## Discussion

Genome integrity is maintained by various mechanisms that either prevent damage to DNA or mediate its repair once the damage has occurred [65]. Inhibition of replication initiation at late-firing or normally dormant origins following DNA damage is one such preventive measure, referred to as the ‘replication checkpoint’ [62]. The exact benefit of late origin inhibition remains enigmatic. For example, it is still unclear whether or not a failure to down-regulate origin firing in the wake of exogenous damage leads to a decrease in genome integrity [66, 67].

**Fig 7:**
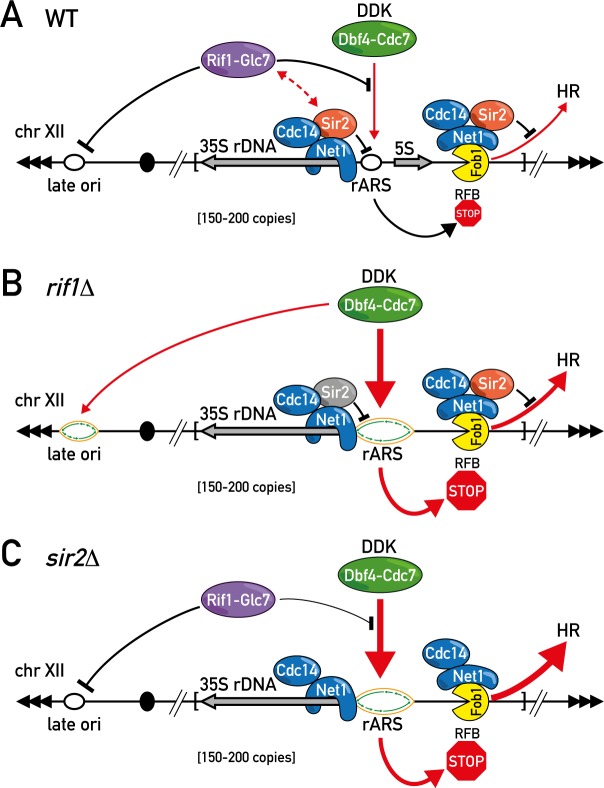
Schematic representation of a model for Rif1 and Sir2 function at the rDNA locus. In WT cells both Rif1-Glc7 and Sir2 inhibit DNA replication initiation at the rDNA locus thereby maintaining its integrity (A). Inactivation of Rif1-Glc7 abolishes the inhibitory dephosphorylation activity and leads to an increase in the DDK-dependent DNA replication initiation at rDNA origins and late origins throughout the genome (B). Deletion of the Sir2 histone deacetylase increases accessibility of the rDNA locus to the limited pool of DNA replication initiation factors (C). Both conditions (B and C) are accompanied by accumulation of replication forks at the Fob1-dependent RFB, leading to rDNA array instability and increased sickness in strain backgrounds deficient in fork maintenance or DNA repair.

Given the DDR-related genetic interactions and phenotypes of budding yeast Rif1 [48, 51] and the recently discovered role of mammalian Rif1 in the DSB repair pathway choice [68] we sought to determine whether yeast Rif1 participates directly in DSB repair, and if so, through what mechanism. To our surprise, we found instead that most of Rif1’s DDR-related phenotypes can be explained by its inhibitory effect on DNA replication within the repetitive rDNA locus and its consequent effect at the rDNA RFB (Fig 7). Our findings suggest that the down-regulation of rDNA replication by Rif1 is important to limit replication fork stalling at RFBs, and presumably a concomitant increase in fork collapse and DNA breakage. In the absence of Rif1, factors involved in either replication fork stabilization or repair (e.g. Mre11, Mms22, Ctf4 and perhaps others) become limiting for survival. This model places Rif1 at the top of a cascade of events leading to DNA damage and impaired growth.

Deletion of *RIF1* or disruption of its interaction with PP1 (Glc7) phosphatase through mutation of the conserved RVxF/SILK motifs led to elevated instability of the rDNA array as detected by the frequency of marker loss from within the array, popping-out of the repeats in the form of ERCs and increased smearing of the chromosome XII on PFGE gels (Figs 3 and S3). Remarkably, the loss of Rif1 did not lead to either a specific increase or decrease in the size of the rDNA array compared to WT (Figs 3D and S3A) but rather to increased variation of the size of the rDNA array in the population of cells, which manifests itself as increased smearing of the chromosome XII band in the PFGE assay (Figs 3D and S3A). This variability may stem from ‘out of register’ repair of damaged rDNA repeats at the same or sister chromatid, from replication fork collapse and re-start on another repeat, or from re-insertion of ERCs into the chromosomal rDNA array. Furthermore, we argue that the *rif1Δ*-dependent increase in rDNA replication is upstream of elevated rDNA instability, since complete alleviation of the latter by removal of the essential causal rDNA fork-blocking factor Fob1 fails to reverse the enhanced rDNA replication in *rif1Δ* cells. Indeed, *fob1Δ rif1Δ* double mutant cells have a fixed rDNA array size (with a sharp chromosome XII band on PFGE), don’t accumulate ERCs, but still exhibit an increase in rDNA replication. These data show that chromosomal (rDNA array) instability and not exclusively that of episomal (ERC) rDNA repeats, are repressed by Rif1-Glc7 inhibition.

Our genetic data place Rif1 in the same pathway as Sir2 with respect to both rDNA replication control and rDNA stability, with the activities of both proteins being essential for inhibition of rARS firing. Based upon the known properties of both proteins, we envisage that Sir2 could restrict accessibility of the rDNA origins to replication initiation factors whilst Rif1-Glc7 decreases their activity by targeted dephosphorylation (e.g. Mcm4 and Sld3, [27]) (Fig 7A). Although both *sir2Δ* and *rif1Δ* deletions lead to more intensive rARS firing, we note an important difference in their effects on the firing of late origins outside of the rDNA. While *sir2Δ* leads to a decrease in the activity of non-rDNA origins, in accordance with models proposing the re-localization of limiting replication factors during S phase [4, 7], *rif1Δ* instead leads to increased firing of non-rDNA origins (Fig 2A and [27]). Our data do not contradict the limiting factors model, as we indeed see some evidence of Sld3 re-localization from an early origin in *rif1Δ* cells (Fig 1C). We imagine that the limiting factors, whose low abundance is unaffected by *rif1Δ* [27], become more active in origin firing in the *rif1Δ* setting due to hyper-activation of DDK and a concomitant increase in CMG DNA helicase activation [27].

Irrespective of the mechanisms of action of Rif1 and Sir2, their inhibition of rARS firing decreases the frequency of stalled forks at RFB and thus DSBs, thereby stabilizing the rDNA repeat locus (Fig 7A). When Rif1 or Sir2 are not present, this replication inhibition is at least partially lost, with a consequent increase of rDNA instability (Fig 7B and 7C). It is also worth noting that Sir2 is loaded onto two locations along the rDNA repeat: at the RFB and near the promoter of the 35S rRNA gene, through interactions with Net1 and its paralog Tof2 [69–71]. As deletion of *FOB1* leads to the loss of Sir2 binding from the RFB site [69], we conclude that the 35S rRNA promoter-bound Sir2 complexes are sufficient to inhibit replication initiation events from the rARS (Figs 3F and 7A). Furthermore, we note that *fob1Δ* had no effect on the *rif1Δ*-dependent increase in BrdU incorporation at the late origins (Fig 3F) and on rDNA replication (Figs 3F and S3D).

At present we do not understand why the *rif1Δ sir2Δ* double mutant displays weaker BrdU incorporation at the rDNA than that of either single mutant. One potential mechanism may involve re-localization of DNA replication factors from rDNA to genomic regions de-repressed only in the *rif1Δ sir2Δ* double mutant and not in single deletions. Further whole-genome replication profiling of the respective mutants would be necessary to address this issue. Alternatively, other factors may come into play when both Rif1 and Sir2 are absent. We note that the Rpd3 deacetylase has been implicated in control of rARS firing [7] and may thus play an unanticipated role in the *rif1Δ sir2Δ* double mutant.

Although both *rif1Δ* and *sir2Δ* mutant cells have elevated numbers of replication intermediates at the rDNA and behave similarly in the BrdU incorporation assay, we noted a marked difference in the relative intensities of bubble and Y arcs in early S phase between *rif1Δ* and *sir2Δ* mutant cells. Namely, *rif1Δ* cells had more bubble arcs and X-shaped molecules, whereas *sir2Δ* and sir2*Δ* rif1*Δ* cells exhibited more prominent Y arcs. We speculate that these patterns may arise due to the repetitive nature of the rDNA array, where the spacing of the activated origins of replication and the rates of fork progression would affect the apparent replication patterns. For instance, one can imagine that the elevated bubble arcs in *rif1Δ* cells may be an indication of either more widely spaced activated origins (therefore the chance of merging of forks early in S phase would be low), or of slower fork progression speed. The increase in Y arcs in *sir2Δ* would fit with previously observed clustering of the activated origins in groups of ca. 2–3 adjacent repeats [6]. Deletion of *SIR2* was shown to increase the sheer number of clusters without disrupting cluster formation. The clustering would lead to faster merging of the forks from adjacent repeats and loss of the bubble arc signal from the 2D gels. We imagine that the clustering of origins might be also beneficial for faster rescue of the forks arrested at the RFBs. Further studies using methods that give spatial resolution, such as DNA combing or electron microscopy would be necessary to investigate the effects of Rif1 and Sir2 on the spacing of origin firing and fork rates at the rDNA array.

Although both *sir2Δ* and *rif1Δ* show a similar increase in rDNA replication initiation, it is interesting to note that Sir2 has a quantitatively larger influence on rDNA stability compared to Rif1. This phenotype has been ascribed to the repressive effect of Sir2 on a bi-directional non-coding promoter (E-pro) located between the 5S rRNA gene and the RFB [22], and it will thus be interesting to determine whether Rif1 also regulates this promoter, with perhaps a weaker effect compared to Sir2. In contrast to its weaker effect on both rDNA silencing and rDNA stability, the Rif1 effect on rDNA replication is very similar in magnitude to that of Sir2, and presumably operates through a different mechanism (Glc7 recruitment versus deacetylation). It is conceivable that the failure to dephosphorylate the previously identified Rif1-Glc7 targets at the origins of replication (i.e. Mcm4 and Sld3) [27] is instrumental in the increased replication and instability of rDNA locus in *rif1* mutants. However, our data do not exclude the possibility that other Rif1-Glc7 dephosphorylation targets exist at the rDNA locus, which could mediate the observed phenotypes. The deacetylation targets of Sir2 relevant for the biology of the rDNA locus also remain poorly explored. Further studies aimed at characterizing known and identifying additional Rif1-Glc7 and Sir2 target proteins should help to shed light on this important question. Another interpretation of our data is that Sir2 is required to recruit Rif1 to its site of action within the rDNA. At present, though, we have no evidence for Rif1 binding within the rDNA from ChIP assays, where we did not detect enrichment above background levels.

The detrimental effect of enhanced replication in a uni-directionally replicated locus may seem paradoxical. Indeed, one can imagine that the elevated number of unimpeded forks at the rDNA in *rif1Δ* and *sir2Δ* cells might be able to efficiently ‘‘rescue” the ones blocked at RFBs, thus neutralizing the effect of the latter, or perhaps even increasing rDNA stability. However, it may also be the case that the proximity of the RFB to the rARS (~ 1.2 kb, in the same rDNA repeat), compared to the nearest possible non-blocked fork (~ 8 kb from rARS in the adjacent rDNA repeat), will mean that forks blocked at RFBs will have to persist for an extended period of time before they can be rescued by a non-blocked fork approaching from the other side. Furthermore, it was shown that both elevated and decreased DNA replication initiation rates at rARS increase rDNA instability [8], and we imagine that some of the DNA replication factors recently shown to be necessary for rDNA stability [72] may act through regulating the replication initiation frequency at rARS, in addition to replisome integrity.

Since Rif1 is recruited to DNA DSBs generated by induction of the HO endonuclease [48] it is possible that Rif1 also participates directly in transactions that occur at accidental DSBs in general, or at DNA breaks that occur specifically at RFB sites. With respect to the former possibility, we note first that *rif1Δ* mutants themselves are not overtly sensitive to DNA damaging agents, and those mutations that display synthetic growth defects in combination with *rif1Δ* (*mre11Δ*, *mms22Δ*, or *ctf4Δ*) so far point to a specific role of Rif1 within the rDNA. However, our data do not rule out a subtle role for Rif1 in repair at all DSBs, and this is a subject worth further investigation. Regarding Rif1’s role within the rDNA, our data point to a specific role for Rif1 Glc7 (PP1) recruitment, and by inference in rDNA replication control. Nevertheless we cannot rule out an additional downstream role of Rif1 in processing breaks generated at the RFB. Finally, we note that the *fob1Δ* background, which would appear to bypass the role of Rif1 in the rDNA stability, may be a valuable tool to study rDNA independent functions of Rif1 in DNA replication and DNA damage response.

Our finding that the effect of budding yeast Rif1 on replication timing is of most consequence at the repetitive rDNA locus may have important implications in more complex eukaryotes where repetitive DNA sequences are much more prevalent. We imagine that there is a strong selection for replication origins within extensive repetitive sequences, and as a consequence mechanisms that help to assure that not all of these identical elements fire within any given cell cycle.

## Materials and Methods

### Genetics and cell growth

All yeast strains described in this study are listed in the S1 Table. General yeast manipulations were done according to standard methods [73]. For the growth assays, overnight cultures of the indicated genotypes were serially diluted 1:10 and spotted onto solid YPAD medium or YPAD medium supplemented with phleomycin (PHL), methyl methanesulfonate (MMS) or camptothecin (CPT). Plates were incubated at the indicated temperature for 2–4 days before being photographed.

The Rif1 anchor away strain (*RIF1-FRB*) was constructed on the basis of the starting strain HHY168, which contains *RPL13A-2XFKB12*, *tor1-1*, and *fpr1Δ*::*NAT* alleles [74], by transformation with a PCR amplified FRB tag substituting the stop codon of the *RIF1* gene. The depletion of Rif1 from the nucleus was achieved by addition of rapamycin (1 μg/ml) to the yeast culture.

### Cell cycle synchronization and DNA polymerase chromatin immunoprecipitation

Cell cycle synchronization for DNA polymerase ChIP-qPCR experiments was achieved as described in [27].

### 5-Bromo-2’-deoxyuridine (BrdU) incorporation and immunoprecipitation

BrdU incorporation was performed as described [75, 76] with minor modifications. Exponentially growing yeast culture arrested in G2/M phase (10 μg/ml nocodazole, US Biological) were pelleted, washed two times and released in fresh media containing 0.2 M hydroxyurea (US Biological) and 400 μg/ml BrdU (Sigma-Aldrich) for 2 hours. Genomic DNA extracted with phenol/chloroform and isopropanol precipitation was sonicated to 500–1000 bp fragments, purified with High Pure PCR Cleanup Kit (Roche) and denatured at 98°C for 5 minutes. Sonicated DNA was immunoprecipitated overnight with 1μg of anti-BrdU antibody (BD Pharmingen) pre-coupled with Dynabeads Protein G (Invitrogen) in IP genomic buffer (1X PBS + 0.0625% Triton X-100). The beads were washed 3 times with IP genomic buffer and once with TE (10 mM Tris-HCl pH8.0, 1 mM EDTA) and DNA was eluted with TE + 1% SDS and purified with High Pure PCR Cleanup Kit (Roche). Immunoprecipitated and input DNA were quantified by qPCR. The BrdU IP is shown as percentage of input or as a relative BrdU incorporation, which is percentage of input of the locus of interest divided by the percentage of input of the negative control (refereed as “ctrl” on Fig 1D) amplifying un-replicated region on chromosome V [7].

### 2D agarose gel electrophoresis

The neutral-neutral 2D agarose gel electrophoreses were performed according to [37] with minor modifications. Briefly, the total genomic DNA from asynchronous cell cultures or cultures released into S phase in the presence of 0.2M HU was isolated with Qiagen Genomic DNA Buffer Set and Genomic-tip. Genomic DNA was digested with *Nhe*I or *Bgl*II, then run in 1^st^ dimension gels (1xTBE; 0.35% agarose) at 50V x 18 hrs. Lanes were excised and run on 2^nd^ dimension gels (1xTBE; 0.9% agarose; 0.3 μg/mL ethidium bromide) at 175V for 13.5 hrs. The resolved DNA was transferred onto nylon membranes, UV cross-linked and hybridized with rDNA probes as described in [77]. The rDNA probe was PCR amplified from W303 yeast genomic DNA, gel-purified and radioactively labeled with Random Primed DNA Labeling Kit (Roche). The images were acquired with FX Personal Phosphorimager (Bio-Rad) and analyzed with Quantity One software. The intensity of the replication intermediates was normalized to the 1n spot and reported as ratio to corresponding replication intermediates in WT strain.

### Extra-chromosomal rDNA circle (ERC) detection

Undigested genomic DNA was resolved on 0.9% agarose gels, transferred onto nylon membranes and hybridized with rDNA probe.

### Telomere southern blot

Telomere southern blot to measure the length of the telomere terminal *Xho*I restriction fragments was performed essentially as described in [27].

### Western blots

Protein extraction by the TCA method and western blotting were performed essentially as described [27]. Rad53 protein was detected (as described in [78]) using Rad53-specific mouse monoclonal antibody raised against total Rad53 protein (Mab clone EL7), or against the active auto-phosphorylated state of Rad53 (Mab clone F9) [79] provided by A. Pellicioli (University of Milan).

### ChIP and qPCR

Chromatin immunoprecipitation of TAP-tagged Fob1 and Sir2 [80] and quantitative PCR were performed as described [27] using Anti-TAP antibody (2 μl per IP, Thermo Scientific) for the immunoprecipitation. For the Sld3-13xMyc and Mcm4-13xMyc ChIP experiments, cells were arrested in G1 with alpha factor prior to the chromatin preparation. Antibody used: anti-Myc, 9E10 from culture supernatant.

### Pulsed-field gel electrophoresis (PFGE)

PFGE was performed as previously described [81] using Bio-Rad DR II Contour-clamped Homogenous Electric Field (CHEF) apparatus. The running conditions were 68 hrs, 12°C, ramping from 300s to 900s switch time. DNA size standards (labeled M in Fig 2D) were purchased from Bio-Rad (*H*. *wingei*, 170–3667). The gel was stained with ethidium bromide, photographed under UV, transferred to a nylon membrane and hybridized with an rDNA probe.

### Marker loss assay for rDNA instability

rDNA instability was measured by the marker loss assay [39, 82]. Saturated yeast cultures where diluted and plated on complete YPD medium supplemented with 5 mg/ml adenine hemisulfate in order to obtain ca. 400 colonies/plate. Plates were sequentially incubated at 30°C (3 days), 4°C (2 days) and 25°C (1 day). The colonies were counted using ImageJ software Colony Counter plugin and the marker loss was calculated as the percentage of white colonies having red sectors. Completely red colonies, representing the progeny of the cells that lost the *ADE2* marker, were excluded from the calculations.

### Statistical analysis

The significance of the difference of the mean values obtained in BrdU IP-qPCR and rDNA instability assays was assessed with two-tailed paired Student’s t-test. The mean and standard error of the mean (SEM) are reported on the graphs.

## Supporting Information

S1 Fig Rif1 inhibits DNA replication initiation at rARS and late origins. (A) Mcm4-13xMyc ChIP at indicated loci in G1-arrested WT and *rif1Δ* cells. (B) Representative FACS profiles from the G2/M arrest and 0.2M HU release experiments. (C) BrdU incorporation at the indicated loci in WT, *rif1Δ* mutant and negative control strains (neg ctrl; a WT strain that lacks the BrdU incorporation cassette). Cultures were released from nocodazole (G2/M) arrest into 0.2 M HU for 2 hrs. Data are presented as mean +/- SEM and a t-test was used to compare the means of WT and mutant cultures. (*) P < 0.05. (D) The schematic representation of the restriction fragments of the rDNA repeats analysed in the 2D agarose gel electrophoreses. (E) 2D gels of *Nhe*I digested genomic DNA from asynchronous cultures probed with rARS probe. Representative images (left panel) and quantification of n = 4 experiments (right panel); the abbreviations are as on the Fig 1E. (F) 2D gels of *Bgl*II digested genomic DNA from asynchronous cultures probed with rDNA RFB probe.
(TIF)

S2 Fig The increase in rDNA instability in *rif1Δ* is not due to DNA damage response or Sir2 re-localization. (A-B) Representative pictures of plates with colonies of strains with indicated genotypes for the rDNA instability assays (*ADE2* marker-loss assay) at Fig 3A–3C. The red sectors on the white colonies are marked with white arrowheads. (C) Exponentially growing cultures of the indicated genotypes were serially diluted 1:10 and spotted onto solid YPAD medium or YPAD medium supplemented with phleomycin (PHL), methyl methanesulfonate (MMS) or camptothecin (CPT). Plates were incubated at 30°C for 3 days before being photographed. The *mre11Δ* mutant serves as a positive control for PHL, MMS and CPT plates. (D) rDNA instability for the indicated WT and mutant strains was measured by the *ADE2* marker-loss assay.
(TIF)

S3 Fig *FOB1* deletion rescues rDNA instability, reverses ERC accumulation but does not affect *rif1Δ* -promoted rARS activation. (A) The chromosomes (chr) from indicated strains were resolved using pulsed-field gel electrophoresis (PFGE) and stained with ethidium bromide (top panel). The same gel was transferred by Southern blot and hybridized with an rDNA probed to detect chr XII (bottom panel). The asterisk marks the position of chr XII in the 20x rDNA strain. (B) ERC accumulation in the indicated strains (see also Fig 3E). (C) PFGE analysis of chr XII heterogeneity (left panel) and 2D gel analysis of rDNA fork pausing (at RFB and elsewhere) in the *rrm3Δ* mutant (right panel; *Nhe*I-digested genomic DNA from asynchronous cells). (D) Deletion of *FOB1* does not alleviate the *rif1Δ* -dependent increase in rDNA replication (2D gels of *Nhe*I-digested genomic DNA from G2/M arrested cultures released in 0.2M HU for 2 hrs).
(TIF)

S4 Fig Telomere length phenotype of *fob1Δ* background and rDNA instability in *rad9Δ* background. (A) Telomere length assayed by Southern blot of *Xho*I-digested genomic DNA in the indicated strains. (B) rDNA instability measured by *ADE2* loss in the indicated strains.
(TIF)

S5 Fig Synthetic sickness of *rif1Δ* with *mre1Δ* is due to rDNA RFBs. Part I. (A–B) Exponentially growing cultures of the indicated genotypes were serially diluted 1:10 and spotted onto solid YPAD media at the indicated temperature supplemented or not with indicated chemicals (PHL, MMS). (C) Tetrad dissection plates of the heterozygous diploids *MRE11*/*mre11Δ* in combination with (left to right): *RIF1/rif1-RBM*; *RIF1/rif1-RVxF/SILK*; *RIF1/rif1Δ*.
(TIF)

S6 Fig Synthetic sickness of rif1Δ with mre1Δ is due to rDNA RFBs. Part II. (A) Tetrad dissection plate of a diploid strain heterozygous for 4 gene deletions: *RIF1/rif1Δ*, *RRM3/rrm3Δ*, *MRE11/mre11Δ*, and *FOB1/fob1Δ* (left panel) and serial dilution spot assays with some of the derived strains (right panel). (B) Serial dilution spot assay of strains harboring combinations of *RIF1*, *SIR2* and *MRE11* gene deletions. (C) Tetrad dissection of a diploid strain with the genotype: *RIF1/rif1Δ*, *SIR4/sir4Δ*, *MRE11/mre11Δ*, *FOB1/fob1Δ*.
(TIF)

S7 Fig Increase in replication initiation in *rif1Δ* leads to elevated DRC. (A) Rad53 phosphorylation upon HU treatment in asynchronous cultures in S288C and JC482 backgrounds (upper panel) and additional rapamycin treatment of WT and *RIF1-FRB* anchor-away strains (both W303, *tor1-1 fpr1Δ RPL13A-2xFKB12*). (B) Rad53 phosphorylation upon HU treatment detected by Western blot in cells harboring *rif1Δ*, combined with *rad9Δ*, *mrc1Δ*, and *sml1Δ mec1Δ* mutations. (C) Asynchronous cell cultures of the indicated genotypes were treated with HU for 2 hours. Subsequently, cells were pelleted, washed and released in fresh media lacking HU, to monitor recovery from the DNA replication checkpoint. Proteins were extracted and Western blotting was performed with antibodies against total Rad53 (Rad53) or the activated (autophosphorylated) protein (Rad53-p), and total Mcm4-13xMyc (which serves as a control).
(TIF)

S1 Table List of yeast strains used in this study. (DOCX)
