# Supplementary figures and images for: Budding Yeast Rif1 Controls Genome Integrity by Inhibiting rDNA Replication
Source: PLoS Genet. 2016 Nov 7;12(11):e1006414. doi: 10.1371/journal.pgen.1006414 (PMC5098799; doi:10.1371/journal.pgen.1006414)

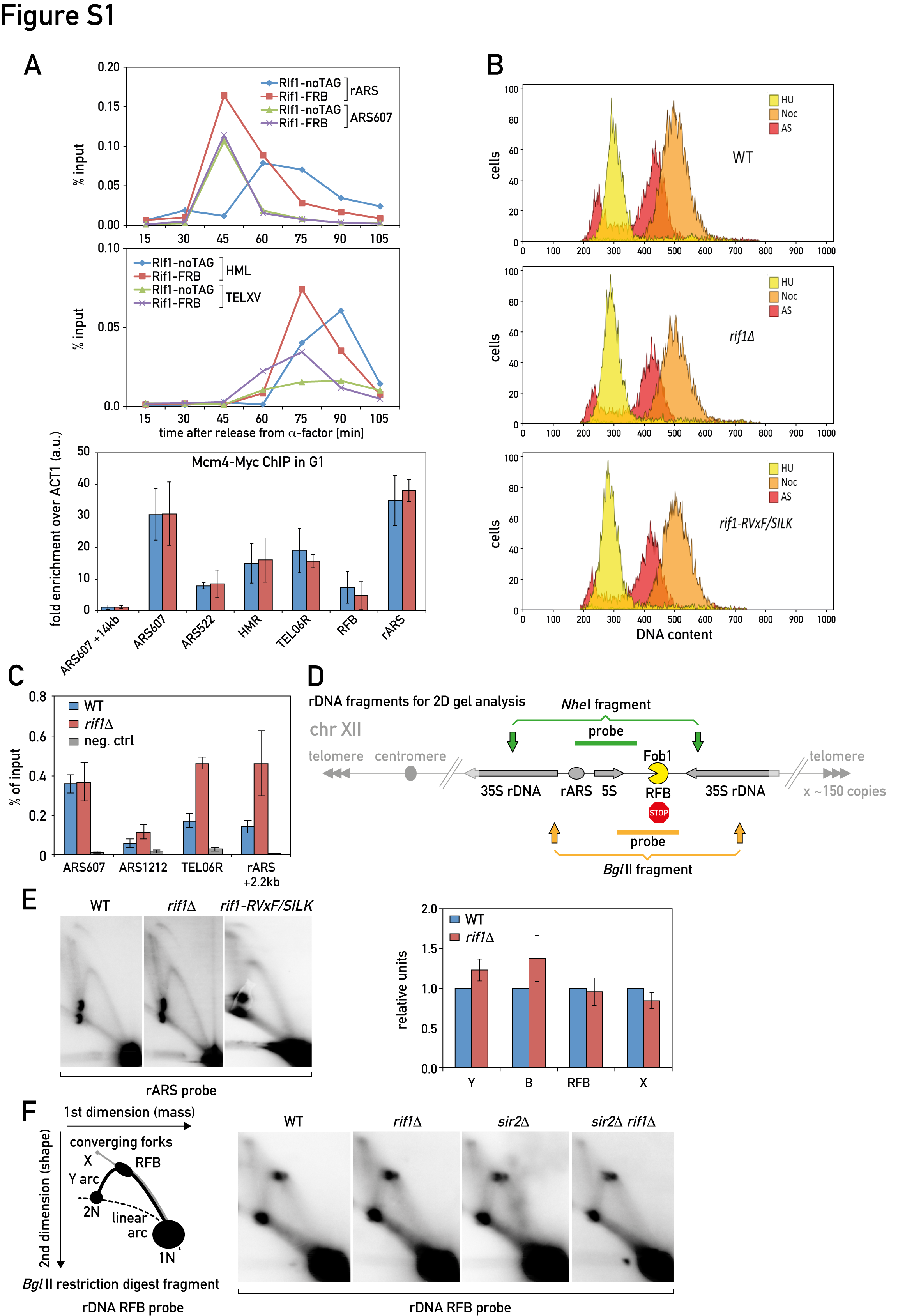

Supplement: S1 Fig — (A) Mcm4-13xMyc ChIP at indicated loci in G1-arrested WT and rif1Δ cells. (B) Representative FACS profiles from the G2/M arrest and 0.2M HU release experiments. (C) BrdU incorporation at the indicated loci in WT, rif1Δ mutant and negative control strains (neg ctrl; a WT strain that lacks the BrdU incorporation cassette). Cultures were released from nocodazole (G2/M) arrest into 0.2 M HU for 2 hrs. Data are presented as mean +/- SEM and a t-test was used to compare the means of WT and mutant cultures. (*) P < 0.05. (D) The schematic representation of the restriction fragments of the rDNA repeats analysed in the 2D agarose gel electrophoreses. (E) 2D gels of NheI digested genomic DNA from asynchronous cultures probed with rARS probe. Representative images (left panel) and quantification of n = 4 experiments (right panel); the abbreviations are as on the Fig 1E. (F) 2D gels of BglII digested genomic DNA from asynchronous cultures probed with rDNA RFB probe. (TIF) [file pgen.1006414.s001.tif]

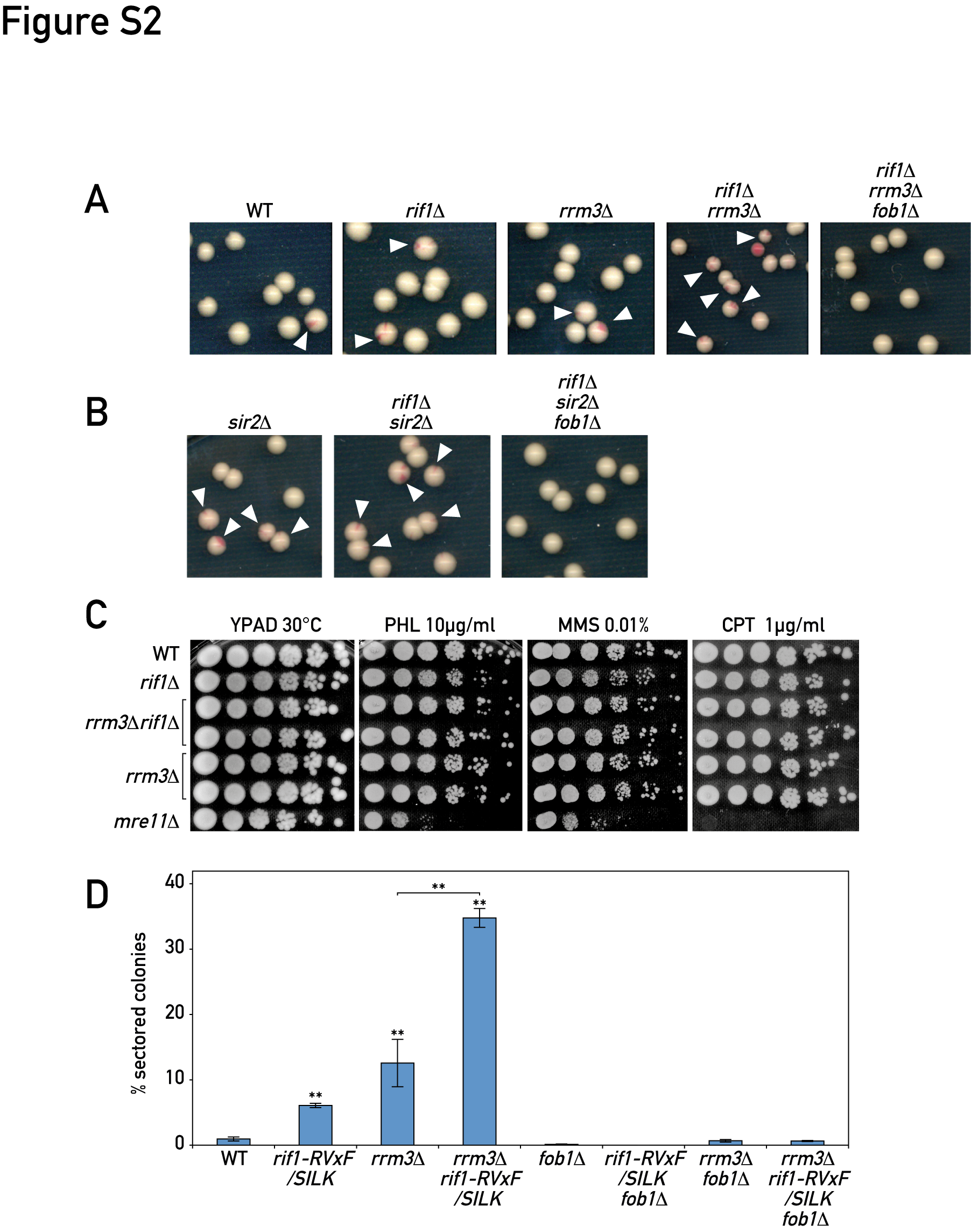

Supplement: S2 Fig — (A-B) Representative pictures of plates with colonies of strains with indicated genotypes for the rDNA instability assays (ADE2 marker-loss assay) at Fig 3A–3C. The red sectors on the white colonies are marked with white arrowheads. (C) Exponentially growing cultures of the indicated genotypes were serially diluted 1:10 and spotted onto solid YPAD medium or YPAD medium supplemented with phleomycin (PHL), methyl methanesulfonate (MMS) or camptothecin (CPT). Plates were incubated at 30°C for 3 days before being photographed. The mre11Δ mutant serves as a positive control for PHL, MMS and CPT plates. (D) rDNA instability for the indicated WT and mutant strains was measured by the ADE2 marker-loss assay. (TIF) [file pgen.1006414.s002.tif]

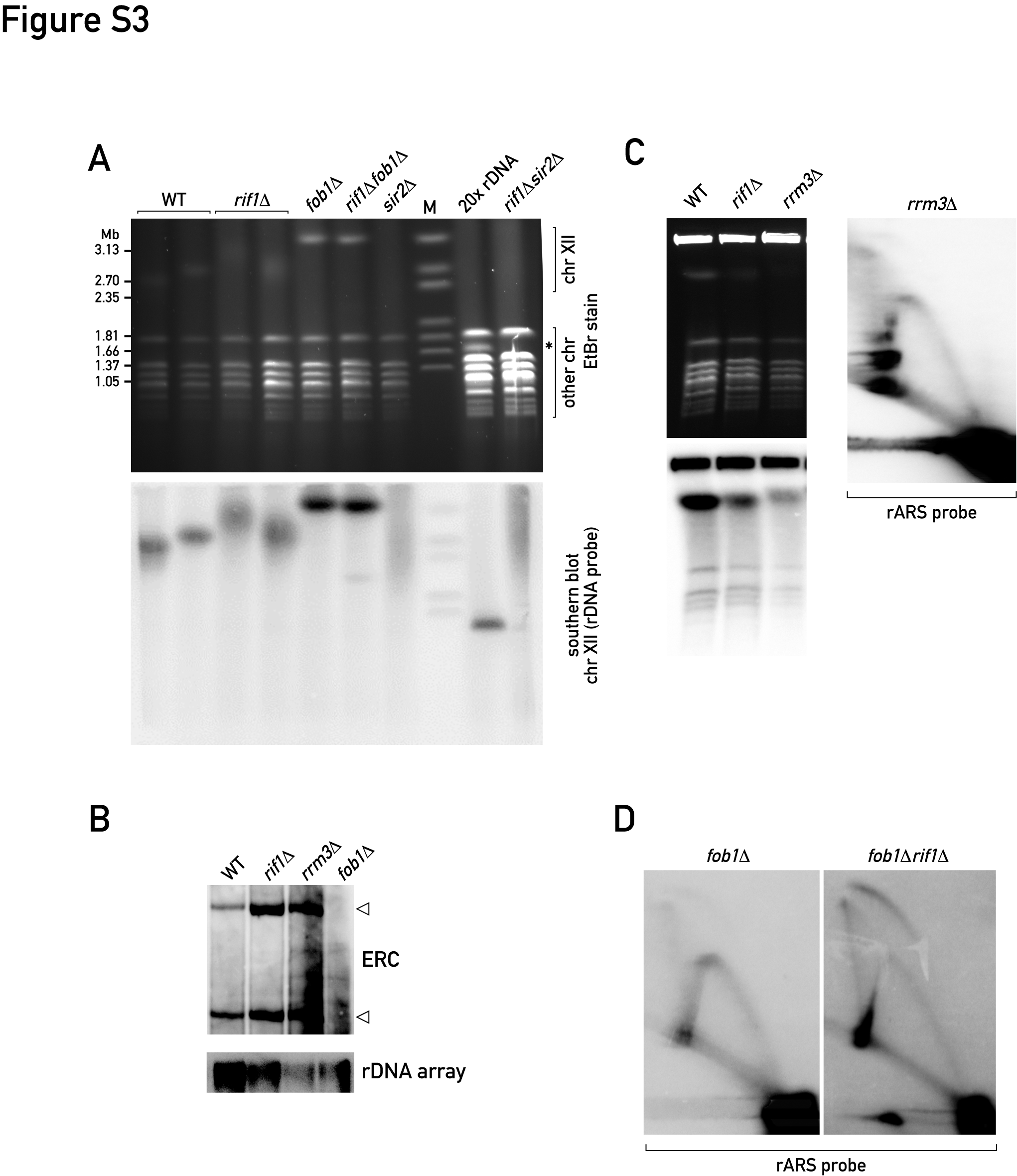

Supplement: S3 Fig — (A) The chromosomes (chr) from indicated strains were resolved using pulsed-field gel electrophoresis (PFGE) and stained with ethidium bromide (top panel). The same gel was transferred by Southern blot and hybridized with an rDNA probed to detect chr XII (bottom panel). The asterisk marks the position of chr XII in the 20x rDNA strain. (B) ERC accumulation in the indicated strains (see also Fig 3E). (C) PFGE analysis of chr XII heterogeneity (left panel) and 2D gel analysis of rDNA fork pausing (at RFB and elsewhere) in the rrm3Δ mutant (right panel; NheI-digested genomic DNA from asynchronous cells). (D) Deletion of FOB1 does not alleviate the rif1Δ -dependent increase in rDNA replication (2D gels of NheI-digested genomic DNA from G2/M arrested cultures released in 0.2M HU for 2 hrs). (TIF) [file pgen.1006414.s003.tif]

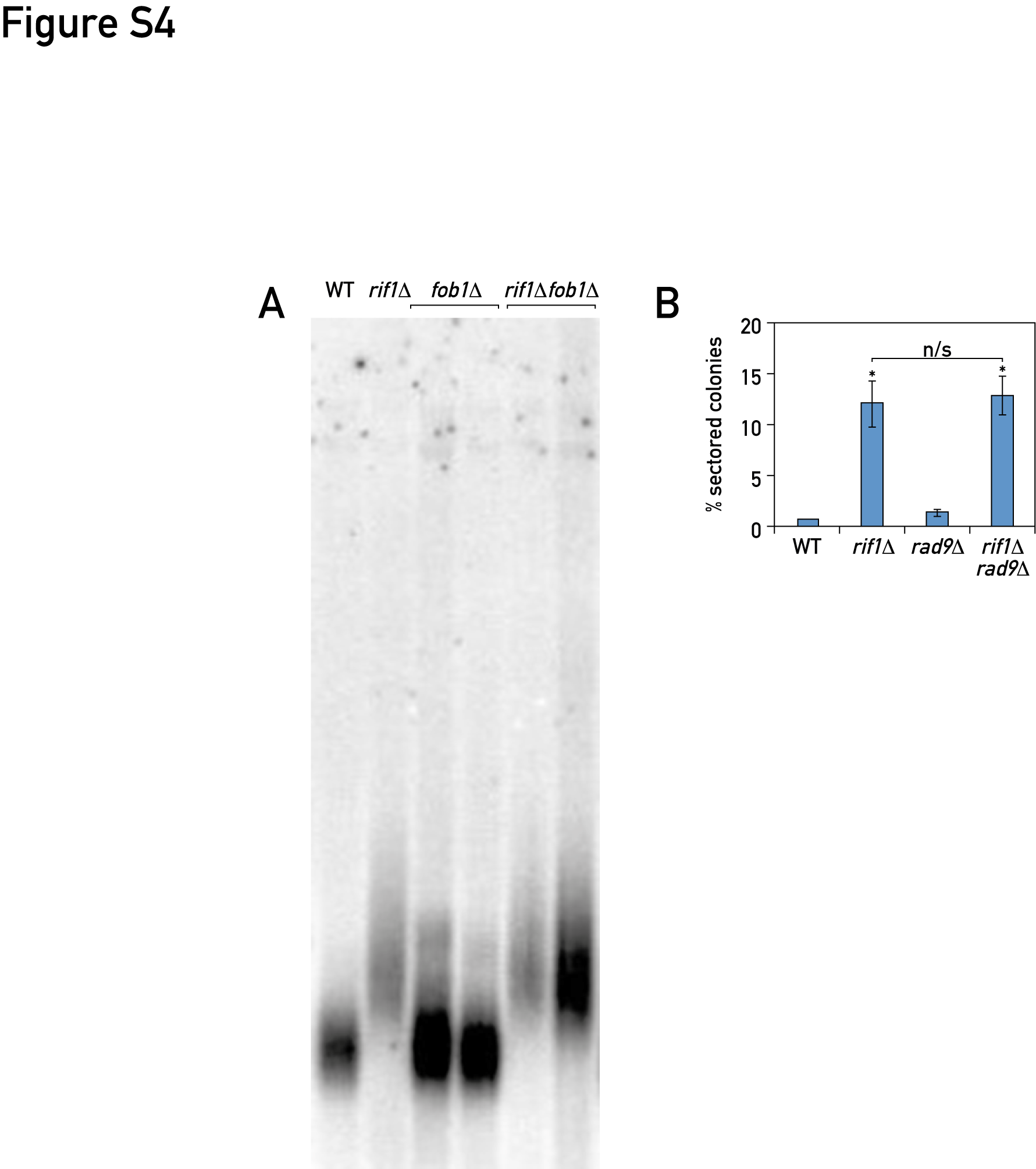

Supplement: S4 Fig — (A) Telomere length assayed by Southern blot of XhoI-digested genomic DNA in the indicated strains. (B) rDNA instability measured by ADE2 loss in the indicated strains. (TIF) [file pgen.1006414.s004.tif]

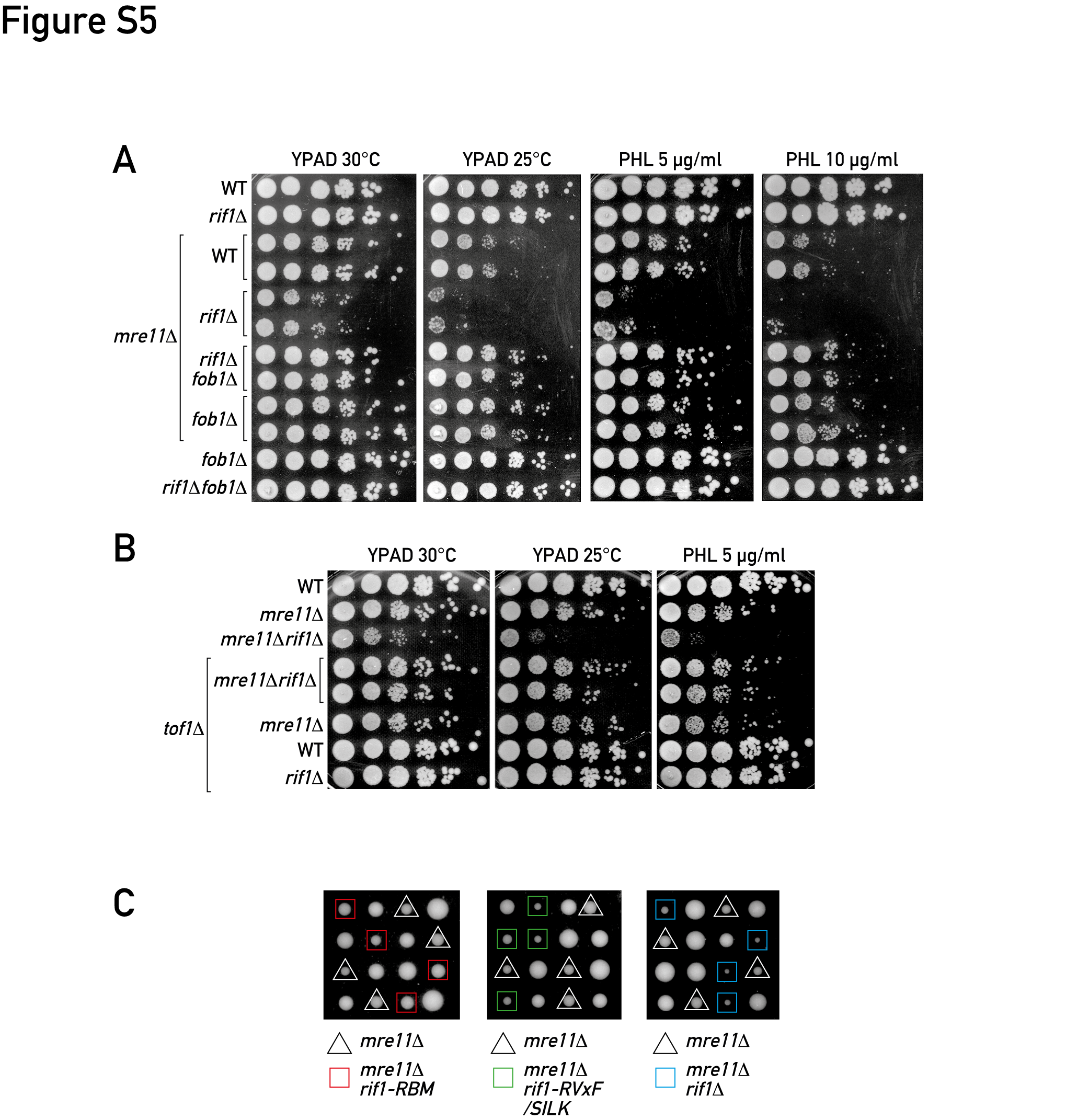

Supplement: S5 Fig — Part I. (A–B) Exponentially growing cultures of the indicated genotypes were serially diluted 1:10 and spotted onto solid YPAD media at the indicated temperature supplemented or not with indicated chemicals (PHL, MMS). (C) Tetrad dissection plates of the heterozygous diploids MRE11/mre11Δ in combination with (left to right): RIF1/rif1-RBM; RIF1/rif1-RVxF/SILK; RIF1/rif1Δ. (TIF) [file pgen.1006414.s005.tif]

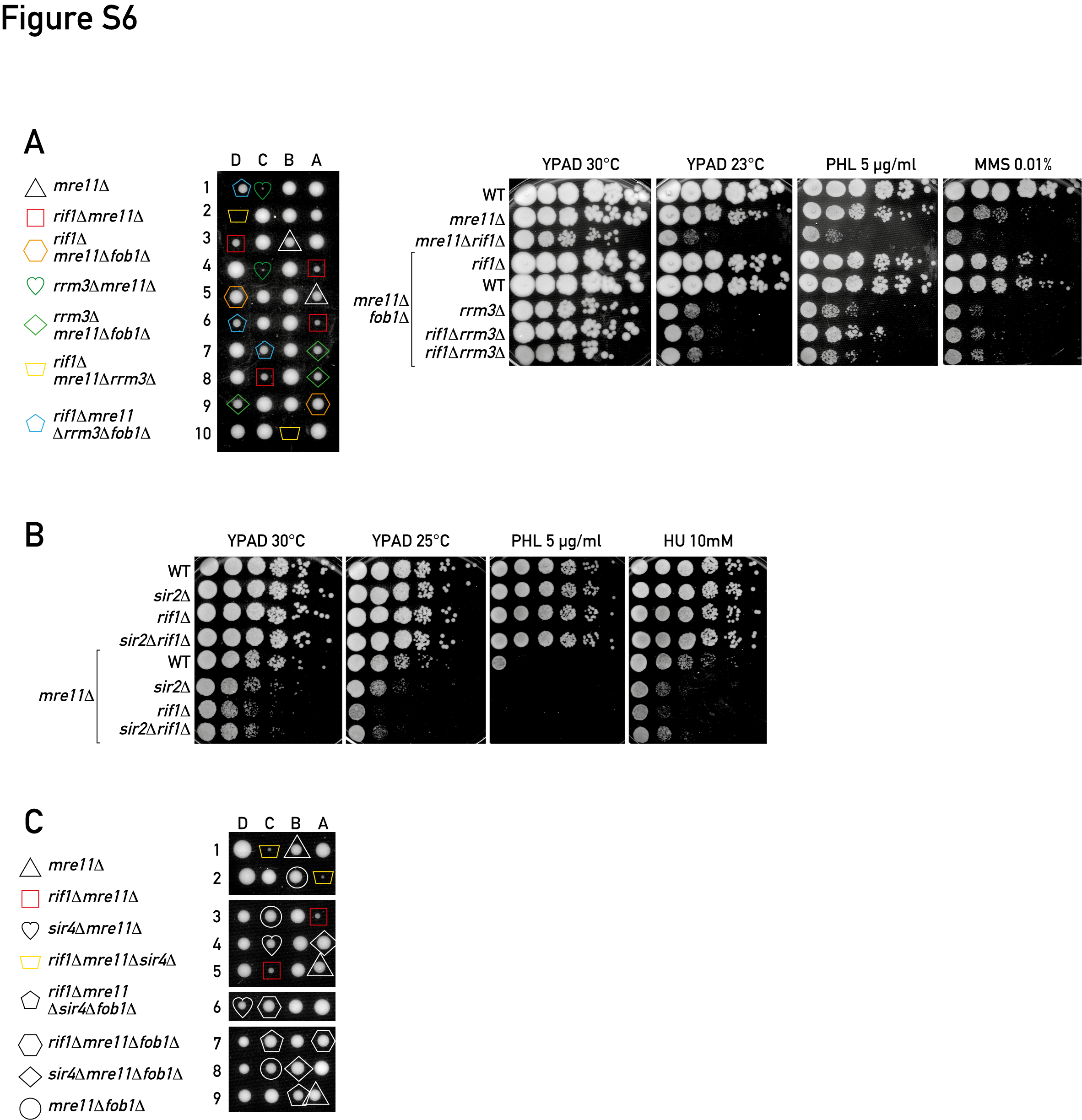

Supplement: S6 Fig — Part II. (A) Tetrad dissection plate of a diploid strain heterozygous for 4 gene deletions: RIF1/rif1Δ, RRM3/rrm3Δ, MRE11/mre11Δ, and FOB1/fob1Δ (left panel) and serial dilution spot assays with some of the derived strains (right panel). (B) Serial dilution spot assay of strains harboring combinations of RIF1, SIR2 and MRE11 gene deletions. (C) Tetrad dissection of a diploid strain with the genotype: RIF1/rif1Δ, SIR4/sir4Δ, MRE11/mre11Δ, FOB1/fob1Δ. (TIF) [file pgen.1006414.s006.tif]

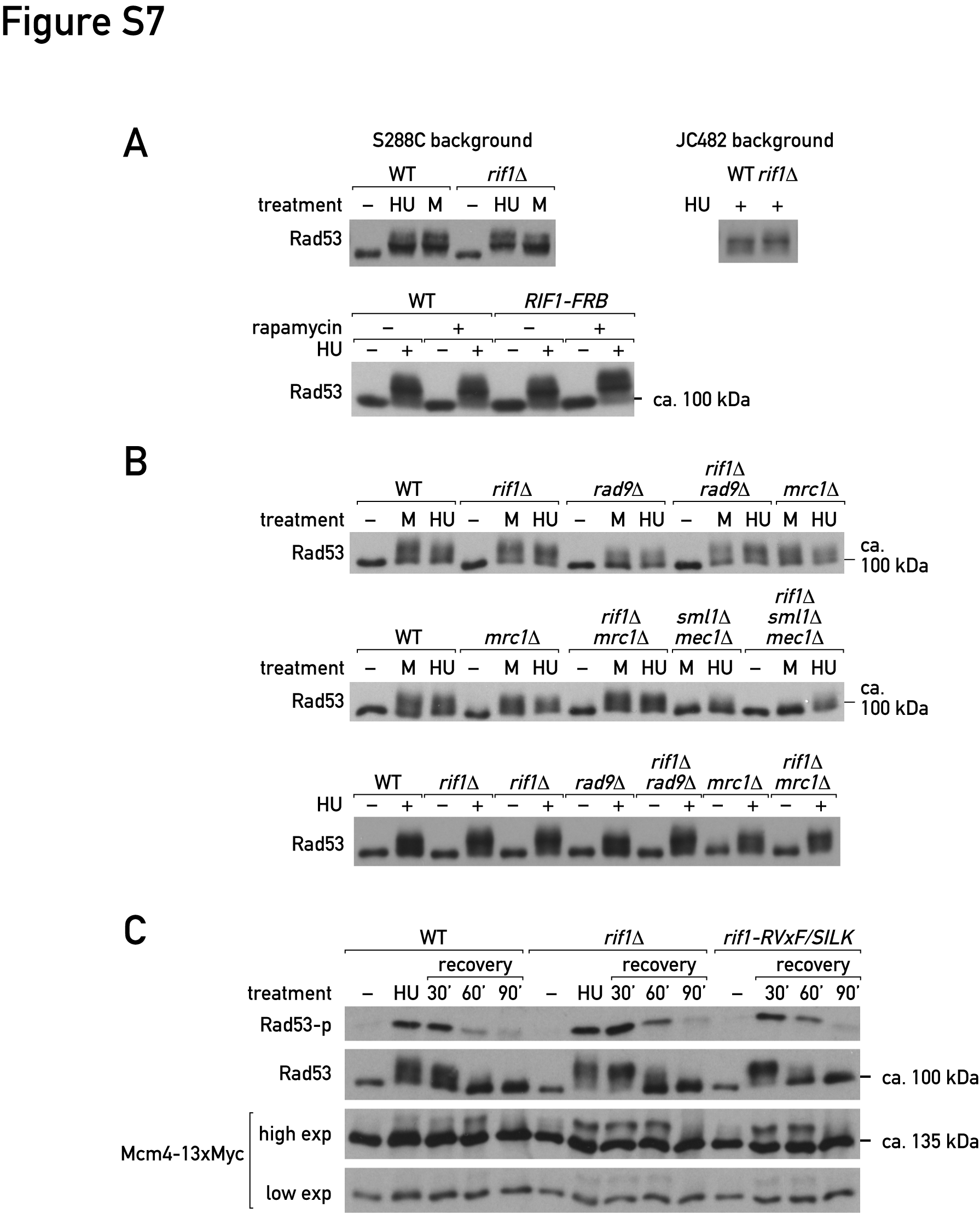

Supplement: S7 Fig — (A) Rad53 phosphorylation upon HU treatment in asynchronous cultures in S288C and JC482 backgrounds (upper panel) and additional rapamycin treatment of WT and RIF1-FRB anchor-away strains (both W303, tor1-1 fpr1Δ RPL13A-2xFKB12). (B) Rad53 phosphorylation upon HU treatment detected by Western blot in cells harboring rif1Δ, combined with rad9Δ, mrc1Δ, and sml1Δ mec1Δ mutations. (C) Asynchronous cell cultures of the indicated genotypes were treated with HU for 2 hours. Subsequently, cells were pelleted, washed and released in fresh media lacking HU, to monitor recovery from the DNA replication checkpoint. Proteins were extracted and Western blotting was performed with antibodies against total Rad53 (Rad53) or the activated (autophosphorylated) protein (Rad53-p), and total Mcm4-13xMyc (which serves as a control). (TIF) [file pgen.1006414.s007.tif]
