# Supplementary material for: Budding Yeast Rif1 Controls Genome Integrity by Inhibiting rDNA Replication
Source: PLoS Genet. 2016 Nov 7;12(11):e1006414. doi: 10.1371/journal.pgen.1006414 (PMC5098799; doi:10.1371/journal.pgen.1006414)
Supplement: S1 Table — (DOCX) [file pgen.1006414.s008.docx]

**S1 Table. Yeast strains used in this study**

| **Strain** | **Relevant genotype** | **Figures** | **Source** |
| --- | --- | --- | --- |
| YDS2 | *MAT*a *leu2-3,112 trp1-1 can1-100 ura3-1 ade2-1 his3-11,15* (W303) |  | Lab collection |
| YLL237 | W303 *MAT*a *RAD5+ bar1Δ FLAG-POL1 POL2-13MYC::KanMX3* | 1B | (Mattarocci et al., 2014) |
| YLL246 | W303 *MAT*a *RAD5+ bar1Δ FLAG-POL1 POL2-13MYC::KanMX3 rif1Δ::NATMX4* | 1B | (Mattarocci et al., 2014) |
| YSM312-1 | W303 *MAT*a *RAD5+ bar1Δ POL2-13MYC::KANMX3 rif1- RVxF/SILK(V116R-F118R-I147R-L148R-R149A)* | 1B | (Mattarocci et al., 2014) |
| YMS1022 | W303 *MAT*a *URA3::BrdU-Inc* | 1D, 1E, 3D, 6C, 6D, S1D, S1E, S3B, S4A | This study |
| YMS1023 | W303 *MAT*a *URA3::BrdU-Inc rif1Δ::NATMX4* | 1D, 1E, 3D, 6C, 6D, S1D, S1E, S3B, S4A | This study |
| YMS1088 | W303 *MAT*a *URA3::BrdU-Inc rif1-RVxF/SILK* | 1D, 3D, S1E | This study |
| YDS2 (neg.ctrl) | W303 *MAT*a | 1D, S1D, 5A, S5 | Lab collection |
| YMS907 | W303 *MAT*a *RDN1::ADE2* | 3A, S2A, S4B, 6B, S3C | This study |
| YMS908 | W303 *MAT*a *RDN1::ADE2 rif1Δ::NATMX4* | 3A, S2A, S4B, 6B, S3C | This study |
| YMS909 | W303 *MAT*a *RDN1::ADE2 rrm3Δ::KANMX6* | 3B, 3E, S2A, S3C | This study |
| YMS910 | W303 *MAT*a *RDN1::ADE2 rif1Δ::NATMX4 rrm3Δ::KANMX6* | 3B, S2A | This study |
| YMS843 | W303 *MAT*a *RDN1::ADE2 rif1Δ::NATMX4 fob1Δ::URA* | 3B | This study |
| YMS911 | W303 *MAT*a *RDN1::ADE2 rrm3Δ::KANMX6 fob1Δ::URA3* | 3B | This study |
| YMS912 | W303 *MAT*a *RDN1::ADE2 rif1Δ::NATMX4 rrm3Δ::KANMX6 fob1Δ::URA3* | 3B, S2A | This study |
| YMS909 | W303 *MAT*a *RDN1::ADE2 rrm3Δ::KANMX6* | 3B, S2A | This study |
| YMS921 | W303 *MAT*a *RDN1::ADE2 RIF1-13xMYC::HIS3* | 4A, 6B | This study |
| YMS922 | W303 *MAT*a *RDN1::ADE2 rif1-rbm(I1762R-I1764R)-13xMYC::HIS3* | 4A, 6B | This study |
| YMS923 | W303 *MAT*a *RDN1::ADE2 rif1- RVxF/SILK-13xMYC::HIS3* | 3B, 6B | This study |
| YMS914 | W303 *MAT*a *RDN1::ADE2 rif1Δ::NATMX4* | 4A | This study |
| YMS915 | W303 *MAT*a *RDN1::ADE2 tel1Δ::KANMX6* | 4A | This study |
| YMS916 | W303 *MAT*a *RDN1::ADE2 rif1Δ::NATMX4 tel1Δ::KANMX6* | 4A | This study |
| YMS918 | W303 *MAT*a *RDN1::ADE2 rif2Δ::KANMX6* | 4A | This study |
| YMS919 | W303 *MAT*a *RDN1::ADE2 rif1Δ::NATMX4 rif2Δ::KANMX6* | 4A | This study |
| YMS930 | W303 *MAT*α *RDN1::ADE2 hmrΔA::TRP1* | 3C, 4B, S3B | This study |
| YMS931 | W303 *MAT*α *RDN1::ADE2 hmrΔA::TRP1 rif1Δ::NAT* | 3C, 4B, S3B | This study |
| YMS932 | W303 *MAT*α *RDN1::ADE2 hmrΔA::TRP1 sir2Δ::KAN* | 3C, S2B, S3B | This study |
| YMS933 | W303 *MAT*α *RDN1::ADE2 hmrΔA::TRP1 rif1Δ::NAT sir2Δ::KAN* | 3C, S2B, S3B | This study |
| YMS934 | W303 *MAT*α *RDN1::ADE2 hmrΔA::TRP1 sir2Δ::KAN fob1Δ::URA3* | 3C, S3B | This study |
| YMS935 | W303 *MAT*α *RDN1::ADE2 hmrΔA::TRP1 rif1Δ::NAT sir2Δ::KAN fob1Δ::URA3* | 3C, S2B, S3B | This study |
| YMS936 | W303 *MAT*α *RDN1::ADE2 fob1Δ::URA3* | 3C | This study |
| YMS1026 | W303 *MAT*α *URA3::BrdU-Inc hmrΔA::TRP1* | 2A, 2B, S1C, 6E, S1F, S3A, S3B | This study |
| YMS1027 | W303 *MAT*α *URA3::BrdU-Inc hmrΔA::TRP1 rif1Δ::NATMX4* | 2A, 2B, S1C, 6E, S1F, S3A, S3B | This study |
| YMS1024-1 | W303 *MAT*a *URA3::BrdU-Inc fob1Δ::KANMX6* | 3F, S3A, 6C, S4A | This study |
| YMS1024-1 | W303 *MAT*a *URA3::BrdU-Inc fob1Δ::KANMX6* | 3D, 3E, 3F, S3B S3D, S4A | This study |
| YMS1025-1 | W303 *MAT*a *URA3::BrdU-Inc fob1Δ::KANMX6 rif1Δ::NATMX4* | 3F , S3A, 6C, S4A | This study |
| YMS1025-2 | W303 *MAT*a *URA3::BrdU-Inc fob1Δ::KANMX6 rif1Δ::NATMX4* | 3D, 3F, S3B, S3D, S4A | This study |
| YMS1028 | W303 *MAT*α  *URA3::BrdU-Inc hmrΔA::TRP1 sir2Δ::KAN* | 2A, 2B,, 6E, S1F, S3A, S3B | This study |
| YSI102 | W303 *MAT*a *20x-rDNA fob1Δ::LEU2* | 6D, S3A | (Ide et al., 2010) |
| YMS1029 | W303 *MAT*α  *URA3::BrdU-Inc hmrΔA::TRP1 sir2Δ::KAN rif1Δ::NATMX4* | 2A, 2B, , 6E, S1F, S3A, S3B | This study |
| YMS330 | W303 *MAT*a *rif1Δ::NATMX4* | 5A, S5 | This study |
| YMS465-1 | W303 *MAT*a *mre11Δ::HPHMX4* | 5A, S5 | This study |
| YMS467-1 | W303 *MAT*a *mre11Δ::HPHMX4 rif1Δ::NATMX4* | 5A, S5 | This study |
| YMS791 | W303 *MAT*a *rif1Δ::NATMX4 mre11Δ::HPHMX4 fob1Δ::KANMX6* | 5A, S5A | This study |
| YMS863 | W303 *MAT*a *rif1Δ::NATMX4 mre11Δ::HPHMX4 tof1Δ::KANMX6* | 5A, S5B | This study |
| YMS867 | W303 *MAT*a *rif1Δ::NATMX4 mre11Δ::HPHMX4 csm3Δ::KANMX6* | 5A | This study |
| YMS741 | W303 *MAT*α *rif1- RVxF/SILK-13xMYC::HIS3MX6* | 5B | This study |
| YMS744 | W303 *MAT*α *rif1- RVxF/SILK-13xMYC::HIS3MX6 mre11Δ::HPHMX4* | 5B | This study |
| YMS1079 | W303 *MAT*a *RAD5+ RIF1-13xMYC::HIS3MX6* | 5C | This study |
| YMS1080-1 | W303 *MAT*a *RAD5+ RIF1-13xMYC::HIS3MX6 mre11Δ::HPHMX4* | 5C | This study |
| YSM1081-1 | W303 *MAT*a *RAD5+ mre11Δ::HPHMX4 rif1Δ::NATMX4* | 5C | This study |
| YMS1082-1 | W303 *MAT*a *RAD5+ mre11Δ::HPHMX4 rif1Δ::NATMX4 cdc7-4* | 5C | This study |
| YMS1083-1 | W303 *MAT*a *RAD5+ rif1Δ::NATMX4 cdc7-4* | 5C | This study |
| YMS883 | W303 *MAT*a *rdn1Δ::2xrDNA pRDN1-hyg::URA3* | 5D, 6D | This study |
| YMS884 | W303 *MAT*a *rdn1Δ::2xrDNA pRDN1-hyg::URA3 rif1Δ::NATMX4* | 5D, 6D | This study |
| YMS885 | W303 *MAT*a *rdn1Δ::2xrDNA pRDN1-hyg::URA3 mre11Δ::TRP1* | 5D | This study |
| YMS886 | W303 *MAT*a *rdn1Δ::2xrDNA pRDN1-hyg::URA3 rif1Δ::NATMX4 mre11Δ::TRP1* | 5D | This study |
| YMS887 | W303 *MAT*a *rdn1Δ::2xrDNA pRDN1-hyg::URA3 rif1Δ::NATMX4 mre11Δ::TRP1 fob1Δ::KANMX6* | 5D | This study |
| YMS888 | W303 *MAT*a *rdn1Δ::2xrDNA pRDN1-hyg::URA3 mre11Δ::TRP1 fob1Δ::KANMX6* | 5D | This study |
| YMS889 | W303 *MAT*a *rdn1Δ::2xrDNA pRDN1-hyg::URA3 rifΔ::NATMX4 fob1Δ::KANMX6* | 5D, 6D | This study |
| YMS890 | W303 *MAT*a *rdn1Δ::2xrDNA pRDN1-hyg::URA3 fob1Δ::KANMX6* | 5D, 6D | This study |
| YMS1035 | W303 *MAT*a/α *RDN1/RDN1::ADE2 HMR/hmrΔA::TRP1 SIR2/sir2::KANMX6 MRE11/mre11Δ::HPHMX4 RIF1/rif1Δ::NATMX4 FOB1/fob1::URA* | 5E | This study |
| YMS1057 | W303 *MAT*a *mms22Δ::HPHMX4* | 5F | This study |
| YMS1058 | W303 *MAT*a *mms22Δ::HPHMX4 rif1Δ::NATMX4* | 5F | This study |
| YMS1059 | W303 *MAT*a *mms22Δ::HPHMX4 rif1Δ::NATMX4 fob1Δ::URA3* | 5F | This study |
| YMS1060 | W303 *MAT*a *mms22Δ::HPHMX4 fob1Δ::URA3* | 5F | This study |
| YMS1061 | W303 *MAT*a *ctf4Δ::KANMX6* | 5F | This study |
| YMS1062 | W303 *MAT*a *ctf4Δ::KANMX6 rif1Δ::NATMX4* | 5F | This study |
| YMS1063 | W303 *MAT*a *ctf4Δ::KANMX6 rif1Δ::NATMX4 fob1Δ::URA3* | 5F | This study |
| YMS1064 | W303 *MAT*a *ctf4Δ::KANMX6 fob1Δ::URA3* | 5F | This study |
| YMS419-4 | W303 *MAT*a *RAD5+ bar1Δ SLD3-13MYC::HIS3MX6* | 1C, S7B | (Mattarocci et al., 2014) |
| YMS438-1 | W303 *MAT*a *RAD5+ bar1Δ SLD3-13MYC::HIS3MX6 rif1Δ::NATMX4* | 1C, S7B | (Mattarocci et al., 2014) |
| YMS781-1 | W303 *MAT*a *RAD5+ bar1Δ SLD3-13MYC::HIS3MX6 rad9Δ::HPHMX4* | S7B | This study |
| YMS782-4 | W303 *MAT*a *RAD5+ bar1Δ SLD3-13MYC::HIS3MX6 rad9Δ::HPHMX4 rif1Δ::NATMX4* | S7B | This study |
| YMS783-3 | W303 *MAT*a *RAD5+ bar1Δ SLD3-13MYC::HIS3MX6 mrc1Δ::HPHMX4* | S7B | This study |
| YMS784-5 | W303 *MAT*a *RAD5+ bar1Δ SLD3-13MYC::HIS3MX6 mrc1Δ::HPHMX4 rif1Δ::NATMX4* | S7B | This study |
| YMS493 | W303 *MAT*a *RAD5+ bar1Δ SLD3-13MYC::HIS3MX6 sml1Δ::HPHMX4 mec1Δ::KANMX6* | S7B | (Mattarocci et al., 2014) |
| YMS494 | W303 *MAT*a *RAD5+ bar1Δ SLD3-13MYC::HIS3MX6 sml1Δ::HPHMX4 mec1Δ::KANMX6 rif1Δ::NATMX4* | S7B | (Mattarocci et al., 2014) |
| YMS1009 | W303 *MAT*a *20x-rDNA fob1Δ::LEU2 rif1Δ::NATMX4* | 6D | This study |
| YMS848 | W303 *MAT*a *RDN1::ADE2 rad9Δ::HIS3MX6* | S4B | This study |
| YMS849 | W303 *MAT*a *RDN1::ADE2 rad9Δ::HIS3MX6 rif1Δ::NATMX4* | S4B | This study |
| YMS973 | S288C *MAT*a *FOB1-TAP::HIS3* | 4C | (Howson et al., 2005) |
| YMS993 | S288C *MAT*a *FOB1-TAP::HIS3 rif1Δ::NATMX4* | 4C | This study |
| YMS972 | S288C *MAT*a *SIR2-TAP::HIS3* | 4C | (Howson et al., 2005) |
| YMS992 | S288C *MAT*a *SIR2-TAP::HIS3 rif1Δ::NATMX4* | 4C | This study |
| YMS790 | W303 *MAT*a *mre11Δ::HPHMX4 fob1Δ::KANMX6* | 5A, S5A | This study |
| YMS860 | W303 *MAT*a *tof1Δ::KANMX6* | S5B | This study |
| YMS862 | W303 *MAT*a *tof1Δ::KANMX6 mre11Δ::HPHMX4* | 5A, S5B | This study |
| YMS861 | W303 *MAT*a *tof1Δ::KANMX6 rif1Δ::NATMX4* | S5B | This study |
| YMS947 | W303 *MAT*a *RDN1::ADE2 rrm3Δ::KANMX6 mre11Δ::HPHMX4 fob1Δ::URA3* | S6A | This study |
| YMS948 | W303 *MAT*a *RDN1::ADE2 rrm3Δ::KANMX6 mre11Δ::HPHMX4 fob1Δ::URA3 rif1Δ::NATMX4* | S6A | This study |
| YMS1047 | W303 *MAT*α *RDN1::ADE2 hmrΔA::TRP1 sir4Δ::LEU2* | 4B | This study |
| YMS1048 | W303 *MAT*α *RDN1::ADE2 hmrΔA::TRP1 sir4Δ::LEU2 rif1Δ::NATMX4* | 4B | This study |
| YMS1049 | W303 *MAT*α *RDN1::ADE2 hmrΔA::TRP1 sir4Δ::LEU2 fob1Δ::URA3* | 4B | This study |
| YMS1050 | W303 *MAT*α *RDN1::ADE2 hmrΔA::TRP1 sir4Δ::LEU2 rif1Δ::NATMX4 fob1Δ::URA3* | 4B | This study |
| YMS871 | W303 *MAT*a/α *RDN1/RDN1::ADE2 HMR/hmrΔA::TRP1 SIR4/sir4::LEU2 MRE11/mre11Δ::HPHMX4 RIF1/rif1Δ::NATMX4 FOB1/fob1::URA* | S6C | This study |
| YMS826-S4C | W303 *MAT*a  *tor1-1 fpr1Δ::NAT RPL13A-2XFKB12::TRP1 bar1Δ::HPHMX4* | S7A | This study |
| YMS825-S3A | W303 *MAT*a  *tor1-1 fpr1Δ::NAT RPL13A-2XFKB12::TRP1 bar1Δ::HPHMX4 RIF1-FRB::KANMX6* | S7A | This study |
| YSM266 | W303 *MAT*a *RAD5+ bar1Δ MCM4-13MYC::HIS3MX6* | S1A, S6C | (Mattarocci et al., 2014) |
| YSM269 | W303 *MAT*a *RAD5+ bar1Δ MCM4-13MYC::HIS3MX6 rif1::NATMX4MX4* | S1A, S6C | (Mattarocci et al., 2014) |
| YSM298 | W303 *MAT*a *RAD5+ bar1Δ MCM4-13MYC::HIS3MX6 rif1- RVxF/SILK* | S6C | (Mattarocci et al., 2014) |
| YSM605 | W303 MATa  *tor1-1 fpr1Δ::NAT RPL13A-2XFKB12::TRP1 bar1Δ::HIS3MX6 POL2-13xMYC::HPHMX4 RIF1-FRB::KANMX6* | S1A | This study |
| YSM606 | W303 MATa  *tor1-1 fpr1Δ::NAT RPL13A-2XFKB12::TRP1 bar1Δ::HIS3MX6 POL2-13xMYC::HPHMX4 RIF1(noTAG)* | S1A | This study |
| YMS1141 | W303 *MAT*a *RDN1::ADE2 RIF1::13xMYC::HIS3* | S2D | This study |
| YMS1142 | W303 *MAT*a *RDN1::ADE2 rif1-RVxF/SILK::13xMYC::HIS3* | S2D | This study |
| YMS1143 | W303 *MAT*a *RDN1::ADE2 RIF1::13xMYC::HIS3 rrm3Δ::KAN* | S2D | This study |
| YMS1144 | W303 *MAT*a *RDN1::ADE2 rif1-RVxF/SILK::13xMYC::HIS3 rrm3Δ::KAN* | S2D | This study |
| YMS1145 | W303 *MAT*a *RDN1::ADE2 RIF1::13xMYC::HIS3*  *fob1Δ::URA3* | S2D | This study |
| YMS1146 | W303 *MAT*a *RDN1::ADE2 rif1-RVxF/SILK::13xMYC::HIS3 fob1Δ::URA3* | S2D | This study |
| YMS1147 | W303 *MAT*a *RDN1::ADE2 RIF1::13xMYC::HIS3 rrm3Δ::KAN fob1Δ::URA3* | S2D | This study |
| YMS1148 | W303 *MAT*a *RDN1::ADE2 rif1-RVxF/SILK::13xMYC::HIS3 rrm3Δ::KAN fob1Δ::URA3* | S2D | This study |
